# The quantitation of buffering action II. Applications of the formal & general approach

**DOI:** 10.1186/1742-4682-2-9

**Published:** 2005-03-16

**Authors:** Bernhard M Schmitt

**Affiliations:** 1 Department of Anatomy, University of Würzburg, 97070 Würzburg, Germany

## Abstract

**Background:**

The paradigm of "buffering" originated in acid-base physiology, but was subsequently extended to other fields and is now used for a wide and diverse set of phenomena. In the preceding article, we have presented a formal and general approach to the quantitation of buffering action. Here, we use that buffering concept for a systematic treatment of selected classical and other buffering phenomena.

**Results:**

H^+ ^buffering by weak acids and "self-buffering" in pure water represent "conservative buffered systems" whose analysis reveals buffering properties that contrast in important aspects from classical textbook descriptions. The buffering of organ perfusion in the face of variable perfusion pressure (also termed "autoregulation") can be treated in terms of "non-conservative buffered systems", the general form of the concept. For the analysis of cytoplasmic Ca^++ ^concentration transients (also termed "muffling"), we develop a related unit that is able to faithfully reflect the time-dependent quantitative aspect of buffering during the pre-steady state period. Steady-state buffering is shown to represent the limiting case of time-dependent muffling, namely for infinitely long time intervals and infinitely small perturbations. Finally, our buffering concept provides a stringent definition of "buffering" on the level of systems and control theory, resulting in four absolute ratio scales for control performance that are suited to measure disturbance rejection and setpoint tracking, and both their static and dynamic aspects.

**Conclusion:**

Our concept of buffering provides a powerful mathematical tool for the quantitation of buffering action in all its appearances.

## Introduction

In the preceding article *(Buffering I *14*)*, we presented a formal and general framework for the quantitation of buffering action. The purpose of the present article is to apply that mathematical tool to the analysis of some scientifically important buffering phenomena.

Recall that we formulated buffering phenomena as the partitioning of a quantity into two complementary compartments, and then used the proportions between the respective flows as a simple quantitative criterion of buffering strength. The two measures of buffering action were *i) *the buffering coefficient b, defined as the differential d(buffered)/d(total), and *ii) *the buffering ratio B, defined as the differential d(buffered)/d(unbuffered). Moreover, the following analyses will make use of the distinction between various categories of buffered systems (e.g. conservative vs. non-conservative partitioned systems), and will exploit the equivalencies and interconversions between these categories.

To begin, we revisit a classical case of acid-base buffering: H^+ ^ion buffering in a solution of a weak acid. This process can be described easily in terms of a conserved quantity (total H^+ ^ions) that partitions into two complementary compartments or states (bound vs. free). Such a system was termed a "conservative buffered system". Conservative buffered systems constitute the most simple buffered systems according to our buffering concept, and they provide a suitable framework to describe further classical buffering phenomena. An important one among them, the so-called "self-buffering" of H^+ ^ions in pure water, is analyzed in Additional file 2.

The concept of a "conservative buffered system" can be applied readily and fruitfully to numerous buffering phenomena that involve quantities other than H^+ ^or Ca^++ ^ions ("non-classical" buffering phenomena). Some examples are presented in the Additional file 3; these include a straightforward approach to the notoriously difficult quantitation of "redox buffering", and examples which demonstrate that the concept of "buffering" is by no means limited to the natural sciences.

In the second section, we analyze the buffering of organ perfusion in the face of variable blood pressure. Here, the independent variable is blood pressure, whereas the dependent variables are volume flows. Systems that involve different physical dimensions, however, cannot be formalized in terms of "conservative buffered systems", the basic form of our buffering concept. Here, the general form of our buffering concept *(Buffering I *14*) *proves to provide a rigorous and reliable framework for the treatment of such "non-conservative" and "dimensionally heterogeneous" buffered systems.

The third section extends the buffering concept to time-dependent buffering processes. "Time" as a potentially important aspect of buffering becomes evident, for instance, in the Ca^++ ^concentration transients that are elicited by the brief openings of a calcium channel in the surrounding cytoplasm [1]. It was an important achievement to realize that this blunting of concentration swings represents an independent quantity, and to suggest a term as fitting as "muffling" for it [2]. However, for reasons detailed in Additional file 5, the available units of "muffling strength" are not satisfying. We introduce an extension of our buffering concept that clearly satisfies all criteria required for a muffling strength unit and provides a dimensionless ratio scale for this quantity. Furthermore, this unit is able to connect "muffling" and "buffering" both conceptually and numerically: Steady-state buffering is shown to represent the limiting case of time-dependent muffling for infinitely long time intervals and infinitely small perturbations.

Finally, Additional file 6 sketches how our concept of buffering can serve to quantitate "systems level buffering" in the context of control systems. Buffering is an important aspect of homeostasis in physiological systems, and control theory provides a powerful general language to describe homeostatic processes. So far, however, the concept of buffering could not be accomodated explicitly in this framework. We show that "buffering" and "control theory" can be connected conceptually and numerically in a straightforward and meaningful way. To quantitate systems level buffering, we need to exploit simultaneously all possibilities and features of our buffering concept, because control systems may be conservative or non-conservative, dimensionally homogeneous or heterogeneous, and time-invariant or time-dependent.

## The buffering of H^+ ^ions by weak acids or bases – Buffering as partitioning of a conserved quantity and the concept of "Langmuir buffering"

Weak acids in conjunction with their conjugate base, and weak bases in conjunction with their conjugate acids, are the prototypical "buffers". They were the first buffers put to action by biochemists in order to stabilize the pH of solutions, and they were also the first buffers to receive thorough theoretical analysis. Numerous textbook definitions explicitly equate "buffers" with "mixtures of weak acids plus conjugate base" (or vice versa), and this notion became so inextricably woven into our thinking about buffering that the distinction between the chemical substrate of this process and the abstract quantitative pattern manifest in it fell into oblivion.

In a wider sense, however, the manifold varieties of ligand binding are indeed responsible for a large number of buffering phenomena encountered in biochemistry or physiology. For instance, ions such as Ca^++ ^are buffered by physico-chemical processes analogous to H^+ ^buffering, albeit without the involvement of literal weak acids or bases. Compared to buffering involving other mechanisms, such as blood pressure buffering or systems level buffering *(see below)*, buffering via ligand binding exhibits some distinct quantitative patterns. The terminology of the original acid-base concept of buffering, however, is too specific as to serve as a general framework for the treatment of these phenomena.

The following section demonstrates how the quantitative patterns of buffering via ligand binding can be caught with the aid of the four parameters t, b, T, and B. The analysis explores the classic case of a weak monoprotic acid dissolved in water.

### Mathematical model of free and bound H^+ ^ion concentrations in a solution of a weak acid

To obtain an explicit quantitative description of buffering by weak acids, we first recapitulate the mathematical model that describes the concentrations of H^+ ^ions in an aqueous solution of a weak acid as a function of *free *H^+ ^ion concentration. Subsequently, we reformulate the concentrations of free and bound H^+ ^ions as functions of *total *H^+ ^ion concentration, and combine these functions into a buffered system. Finally, we derive the four parameters t, b, T, and B from this system.

A weak monoprotic acid HA can dissociate into a free H^+ ^ion and a conjugated base A^-^, to an extent that is dictated by K_A_, the acid constant in water, according to:

K_A _× [HA] = [H^+^]_free _× [A^-^]_free_.

The total amount of weak acid [A]_total _equals the sum [A^-^]_free_+ [HA] of dissociated and undissociated weak acid; the system is "conservative". We can therefore substitute [A^-^]_free _by [A]_total_- [HA] and obtain:



and after several intermediate steps:



For the sake of readability, we express the same relationship in a more general notation:



where c and d stand for the constants [A]_total _and K_A_, respectively, and the variables y and z correspond to [H^+^]_free _and [HA], respectively. This latter equation describes a hyperbola that approaches c as y increases to infinity. It was first used empirically by Hill [3], but became a much more meaningful mathematical model after Langmuir had supplied a mechanistical interpretation, namely in terms of non-cooperative binding of a ligand to a finite number of binding sites [4]. That model is widely applicable to numerous phenomena, e.g. receptor-ligand interactions, adsorption processes at surfaces, or enzyme kinetics, to name but a few. The same rules of non-cooperative binding apply to the binding of H^+ ^ions to the conjugate base of a monoprotic weak acid.

In order to move from specific acid-base terminology to a general ligand binding terminology, we re-interpret the symbols in the equation  as follows: Variable z represents the concentration of bound ligand, y the concentration of free ligand, c the total number of binding sites, and d the equilibrium constant K_d _of the complex with respect its dissociation products.

### "Langmuir buffers"

**Figure 1:**
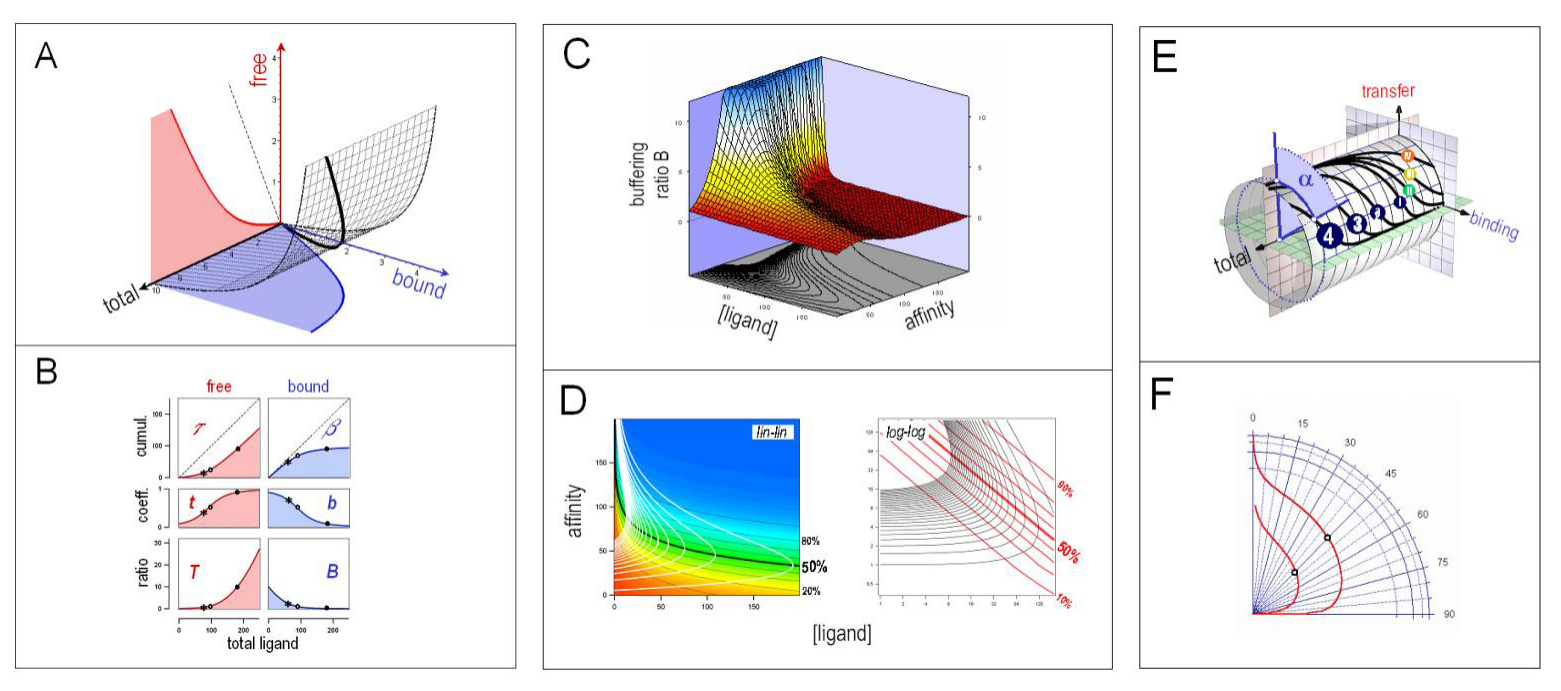
***Buffering via non-cooperative ligand binding: "Langmuir buffering"***. The prototype of Langmuir buffering is the buffering of H^+ ^ions in a solution of a weak acid. ***A, Relation between the three variables of a "Langmuir"-type buffer***. Concentrations of free ligand *(red)*, bound ligand *(blue)*, and total ligand in a solution of a weak acid. The relations between the three variables are computed from the equation , where K_d _stands for the dissociation constant of the buffer-ligand complex, and [buffer] for total buffer concentration. [buffer] and K_d _are assumed to be constant. Plotted for [buffer] = 5 and K_d _= 1. ***B, Describing "Langmuir buffering" using the four buffering measures t, b, T, and B***. Titration of a "Langmuir buffer" with increasing concentrations of ligand; constant parameters are: [buffer] = 100, K_d _= 10, arbitrary concentration units. Characteristic system states shown are the "half-saturation point" of buffer *(asterisk)*, the "equipartitioning point" where half of the added ligand remains free, and the other half is bound by the buffer *(open circle)*, and the "break even point" where the ligand inside the system is half bound, half free *(closed circle)*. *Top panel, left: *Transfer function τ, i.e., free ligand concentration *(ordinate) *as a function of total ligand *(ordinate*). *Top panel, right: *buffering function β, i.e., bound ligand concentration as a function of total ligand. *Middle panel, left: *Transfer coefficient *t*, i.e., the (differential) fraction of added ligand that enters the pool of free ligand. *Middle panel, right: *Buffering coefficient *b*, i.e., the (differential) fraction of added ligand that becomes bound to buffer. *Bottom panel, left: *Transfer ratio *T *= *d*(free)/*d*(bound), i.e., the differential ratio of additional free ligand over additional bound ligand. *Bottom panel, right: *Buffering ratio *B *= *d*(bound)/*d*(free), i.e., the differential ratio of additional bound ligand over additional free ligand. The parameters b and B provide two complementary measures of buffering strength. ***C, Buffering strength of a Langmuir buffer as a function of both total ligand concentration and affinity***. *Wireframe surface: *The buffering ratio B is shown on the vertical axis; affinity expressed as 1/K_d_; concentration of ligand, [ligand], and K_d _in arbitrary concentration units. *Contours on bottom: *Lines connect states of identical buffering strength. For a buffer with a given K_d_, buffering strength decreases monotonically with increasing ligand concentration. However, at a fixed ligand concentration, buffering strength as a function of affinity runs through a maximum. ***D, Visualizing Langmuir buffering by two-dimensional plots (same data as in Figure C)***. *Left hand*, linear plot; *white lines*, states of identical buffering strength; *black lines*, states of identical fractional buffer saturation. *Right*, double-logarithmical plot. *black lines*, states of identical buffering strength; *red lines*, states of identical fractional buffer saturation. ***E, Using the "buffering angle" to visualize Langmuir buffering: cylinder plot***. As shown in *Buffering I*, the specific angle α for which [α = arccos(T) and α = arctan(B)] can unambiguously represent the buffering parameters t(x), b(x), T(x) and B(x) at a given point on the x axis. Consequently, a curve on the surface of a unit cylinder can represent the buffering behavior for an entire range of x values, yielding a "state portrait". State portraits of several Langmuir buffers are shown. *Curves with Roman numerals (I-IV) of different color*: effect of decreasing ligand affinity at fixed total concentration. *Curves with Arabic numerals (1–4) of different size*: effect of increasing total buffer concentration. Less intuitively, yet more practically, the cylinder surface may be "flattened" out and represented in two dimensions *(not shown)*. *Blue segment: *buffering angle α for curve 4. ***F, Using the "buffering angle" to visualize Langmuir buffering: polar graph***. Alternative form of a buffering state portrait: each point on the curve is characterized by a "buffering angle α " with the vertical axis (clockwise) and a radius (here plotted logarithmically), which correspond to buffering angle α and total ligand concentration, respectively. *Open circles*, equipartitioning points, i.e., where t = b and α = 45°.

So far, we have described bound ligand z as a function of free ligand y. Next, we express the concentration of free ligand y as a function of total ligand x = y+z:



and the concentration of bound ligand z as a function of total ligand x:



The relation between total, free, and bound ligand for non-cooperative binding to a fixed number of binding sites with similar affinity is shown in Figure 1A. It is easy to double-check that the sum y(x)+z(x) equals x, i.e., the conservation condition is satisfied.

To turn these two functions into a "buffered system", we assign the role of "transfer function" τ(x) to the free H^+ ^concentration y(x), and the role of "buffering function" β(x) to the bound H^+ ^concentration z(x). Because many common and important systems follow this quantitative pattern, it might be useful to have a specific term to refer to them. We suggest the term *"Langmuir-type buffers" *or *"Langmuir-type buffered systems"*. Briefly, a Langmuir-type system can be defined as an ordered pair of two functions {y = τ(x), z = β(x)} that satisfies the three conditions (x = y+z) and z = c × y/(d+y) and c, d ∈ ^+^.

In systems that involve ion concentrations, both τ(x) and β(x) naturally assume a value of zero at x = 0, i.e., they pass through the origin. However, we can relax this fourth constraint by allowing for offsets τ_0 _and β_0_, respectively, without altering the buffering properties (Buffering I 14). Thus, we obtain the general form of a Langmuir buffer B_Langmuir _as:



The buffered system constituted by the solution of a weak acid in water is "dimensionally homogeneous": the variables x, y, and z are either all dimensionless (e.g. when expressed as multiples of K_d _or K_A_), or they all have the dimension of a concentration (e.g. when expressed in moles/liter). Similarly, H^+ ^buffering in pure water is represented by a dimensionally homogeneous buffered system (Additional file 2).

### Computing the buffering parameters in Langmuir-type systems

Because we find in conservative systems that τ'(x)+β'(x) = 1, the transfer and buffering coefficients are simply equal to the respective first derivatives:



These equations have unique solutions as long as the constants c and d are positive; for dissociation constants and concentration terms, this is always warranted. It is easy to verify that, consistent with the conservation condition σ'(x) = 1, the coefficients t and b always add up unity.

From t and b, we can then compute the transfer ratio T and the buffering ratio B as functions of x:



Expression of transfer and buffering ratio as functions of y (instead of x) results in equivalent, yet much simpler forms:



The buffering parameters t, b, T, and B provide a complete description of H^+ ^buffering by weak acids (Figure 1B). They allow us to elucidate the common properties of all Langmuir buffers, both in terms of a communicating-vessels model (Additional file 1) and mathematically *(see following paragraph)*.

#### General properties of Langmuir buffer systems

##### Langmuir buffers are "finite capacity buffers"

At x = 0, the buffering function β(x) has a finite value β_0 _∈ R. As x increases, β(x) increases monotonically, asymptotically approaching a finite value c+β_0_. When a Langmuir buffer is modelled by communicating vessels, the buffering vessel has a finite volume, in spite of its infinite height.

##### Buffering strength is maximal when ligand concentration is zero

In absolute values, we find for buffering coeffient b and buffering ratio B at ligand concentration x = 0:



and



The corresponding values of transfer coefficient t and transfer ratio T are:



and



In the model, the cross-sectional area of the buffering vessel is largest at its base.

For the special case of H^+ ^buffering in a solution of a weak acid, this means: The maximum buffering ratio B is obtained simply by divding the concentration of total weak acid by the acid constant K_A_:



This relationship can be exploited to elegantly determine total concentration A_total _of a buffer with known K_A _or K_d_: The buffering ratio B is determined experimentally at ligand concentrations that are much smaller than K_d _(x<<K_d_), from which A_Tot _can be approximated as A_total_≈B × K_d _[5].

##### Langmuir buffers are "non-linear buffers"

Buffering coefficient b and buffering ratio B decrease monotonically with increasing x (or y), asymptotically approaching zero. In the communicating vessels-model, the buffering vessel is not parallel-walled, but tapers off towards the upper end.

##### Langmuir buffers are "non-inverting moderators"

Over the entire domain ^+^, the buffering coefficient b assumes values between 0 and 1 (0≤b<1), and the buffering ratio B is always nonnegative (B≥0). In the model, this property is apparent inasmuch as the buffering vessel has fixed walls with positive-valued cross-sectional areas (in fact, "negative-valued cross-sectional areas" do not exist, and the vessel model can thus not replicate amplification or inversion).

##### Langmuir buffers have a "break even point" at x = 2c-2d

In the vessel model, "break even points" are fluid levels at which transfer and buffering vessel each contain identical fluid volumes. Trivially, this is the case when both vessels are empty, or at x = 0. However, there is a second such system state at x = 2(c-d) if c>d (based on the definition c, d>0 and assuming that both functions cross the origin). For x<2(c-d), the greater part of the quantity is found in the "buffering compartment"; for x>2(c-d), the greater part is in the "transfer compartment". *"Break even-point" *may be a suitable term to refer to this point. If however c<d, then no second break even-point exists, and the transfer compartment contains at all values of x more of the quantity than the buffering compartment.

##### Langmuir buffers have a half-saturation point at x = c/2+d

In the vessel model, half-saturation of buffer means that the buffering vessel is half full. In terms of total volume x, there is a value x_0.5 _for which the buffering function β(x) becomes equal to , namely at , assuming that τ_0_,β_0 _= 0. Thus, x_0.5 _may be called the *"half-saturation point" *of a given Langmuir-buffer. In terms of the the transfer function y = τ(x), half-saturation of a Langmuir buffer is reached at y = d. This result is a well known property of systems conforming to Langmuir's equation. Naturally, *infinite *capacity-buffers (e.g. pure water which is not a Langmuir buffer) cannot have a half-saturation point.

##### At half-saturation, the buffering ratio B of a Langmuir buffer is one fourth of its maximum

When the buffering vessel is half full, its cross-sectional area is one fourth of the cross-sectional area of the transfer vessel. At the half-saturation point x_0.5_, buffering strength has the following values:



and



Thus, for H^+ ^buffering in a solution of a weak acid, the buffering ratio B at half-saturation is one fourth of the concentration of total weak acid divided by the acid constant: [A]_total_/(4 × K_A_).

##### Langmuir buffers have an "equipartitioning point" at x = c-d

In the vessel model, equipartitioning means that the partial flows into the two partitions (buffering and transfer compartment) are equal. This is the case when transfer and buffering vessel have equal cross-sectional areas. Here we find that b = t = 0.5 ∧ B = T = 1. Note the difference between "break even point" and "equipartitioning point".

### Comparison with other descriptions of H^+^-buffering by weak acids

Analysis of H^+ ^buffering by weak acids by means of the buffering coefficient b and buffering ratio B has thus led to conclusions that differ considerably from the standard view of buffering which is based on Van Slyke's "buffer value". Interestingly, our units will, similarly to Van Slyke's buffer value, identify as the strongest H^+ ^ion buffer for a given pH value that weak acid whose pK_A _equals this pH, in spite of the conflicting conclusions as to the point of maximum buffering strength. Additional file 1 discusses in more detail the impact of different units on our perception of H^+ ^buffering by weak acids or bases.

### Other conservative buffered systems

This "worked example" of H^+ ^buffering by weak acids demonstrated how the concept of conservative buffered systems can be applied in practice. There are multiple other buffering phenomena that conform to that concept and which can be analyzed in exactly the same manner. Among them, H^+ ^buffering by pure water is of particular interest (Additional file 2). Oxygen buffering by hemoglobin, a mechanism of great physiological importance, would be another example of the same basic type, but not involving H^+ ^ions, and with yet different quantitative behavior. The concept of conservative buffered systems can also be applied directly to quantities that are governed by mechanisms unrelated to ligand binding, e.g. to heat energy. Moreover, conservative systems need not be restrained to non-inverting moderation, but may exhibit amplification as well. Such non-classical examples of conservative buffered systems are presented in Additional file 3. Non-conservative systems are treated in the following section.

## The buffering of organ perfusion in the face of blood pressure fluctuations – The concept of "nonconservative buffered systems"

### The term "blood pressure buffering" is used in various ways

Some use this term as a synonym to "autoregulation" of organ perfusion, i.e., the maintenance of a constant blood flow in the face of variable blood pressure or cardiac output. Others apply it to the mechanisms that blunt the pressure-raising or -lowering effects of physiological maneuvers and drugs; according to a major contributor, this phenomenon is also called "baroreceptor buffering" [6,7]. Finally, the term "blood pressure buffering" sometimes refers to the attenuation of blood pressure *variability*, i.e., of the oscillations of mean arterial blood pressure (MAP) around its average [8-10].

### Lack of a quantitative measure of "blood pressure buffering"

Experimental studies on blood pressure buffering usually report the magnitude of all relevant basic quantities in terms of scientific scales. In contrast, the magnitude of "blood pressure buffering" itself is specified merely in terms of "more" or "less", without attempting to extract from the data some specific numerical value that could serve as a measure of this central quantity. In other words, the currently available scales for blood pressure buffering strength are non-metrical, ordinal scales. These scales are rather primitive and do not allow one to carry out a number of desirable and legitimate scientific operations (e.g. mutual comparisons of the buffering strengths of individual mechanisms that jointly contribute to blood pressure buffering, or comparison of "blood pressure buffering" to the buffering of other physiologic parameters such as pH, Ca^++ ^concentration, or body temperature).

This section demonstrates that our concept of buffering readily quantitates "blood pressure buffering" in most of its meanings. However, many of these phenomena cannot be described any more in terms of simple "conservative" partitioned systems. Rather, the systematic treatment of these buffering phenomena makes it necessary to recall and utilize the distinctions between various categories of buffered systems that were outlined in the preceding article *(Buffering I *14*)*: conservative vs. non-conservative, and dimensionally homogeneous vs. heterogeneous systems. Moreover, one type of blood pressure buffering, that of blood pressure *variability *buffering, will turn out to resist formalization as a "buffered system" altogether, suggesting that this paradigm actually refers to something that is essentially different from buffering in the common sense.

### Autoregulation of flow in the face of variable total flow – dimensionally homogeneous systems (Figure 2A–C)

**Figure 2:**
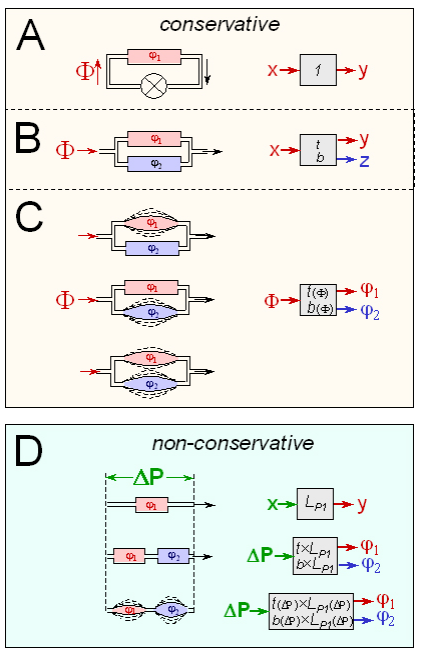
***Buffering in conservative and non-conservative buffered systems, illustrated by blood pressure buffering***. ***A-C, Conservative buffered system: Buffering individual flow against variations of total flow***. Pipework model of circulation, where cardiac output corresponds to total volume flow Φ, and volume flows in individual organs to volume flows φ_i _in individual tubes. ***A, Zero buffering***. In a circulation comprising a single hydraulic conductor, a total volume flow (Φ) established by a pump (⊗) results in a partial flow (φ_1_) of equal magnitude in the conductor *(red)*. Their quantitative relation can be represented in signal transduction formalism as a "transfer element" where input x corresponds to Φ, output y to φ_1_, and the transfer properties are characterized by a constant transfer coefficient of 1. ***B, Linear buffering***. Total volume flow partitioning into two parallel hydraulic conductors. Changes of total flow Φ now elicit smaller changes of the partial flow φ_1 _*(red) *– due to "buffering" by the second conductor *(blue)*. Transfer and buffering behavior with respect to the upper vessel can be expressed in terms of fixed, dimensionless fractions t and b. ***C, Nonlinear buffering***. When one or both vessels have elastic walls, hydraulic conductance and thus responsiveness to changes in Φ will vary with the absolute value of Φ. Transfer and buffering coefficients become nonlinear functions of Φ. ***D, Non-conservative buffered systems: Buffering individual flow against variations of perfusion pressure***. Organ volume flows φ_i _are described as functions of perfusion pressure ΔP. With different physical dimensions for input and output (pressure vs. flow), the transfer coefficient for vessel 1 alone has the dimension of a hydraulic conducance L_P1_. With a second vessel added in series, changes of perfusion pressure translate into smaller changes of volume flow. This effect can be interpreted as "buffering" and expressed quantitatively using the buffering parameters t, b, T, and B. If one or both vessels are elastic, transfer and buffering functions become nonlinear functions of perfusion pressure ΔP.

We can describe the individual volume flows in several parallel tubes as a function of total volume flow through that system. We then find that flow in a given tube is stabilized or "buffered" against a given change of total flow by the parallel tubes. This is one way how one can achieve stabilization of organ perfusion in the face of variable blood flow (e.g. at rest vs. exercise), or its adaptive regulation (e.g. in the skin, via opening or closing of shunting vessels). Such systems can be formalized as conservative buffered system and analyzed in the same way as shown for H^+ ^buffering *(see above) *and for other phenomena (Additional file 3; there, this particular case is also worked out explicitly).

### Autoregulation of flow in the face of variable pressure – dimensionally heterogeneous buffered systems (Figure 2D)

#### Perfusion in the absence of autoregulation or buffering

Rather than using total volume flow as independent variable, volume flows in systems of tubes may be expressed as functions of pressure. According to the basic law of convective volume transport, the flow Φ in a single tube A is a linear function Φ = L_A _× ΔP of the pressure difference ΔP between its inlet and outlet, with hydraulic conductance L_A _as the proportionality factor. Assumptions herein are laminar flow and the absence of other relevant forces such as osmotic gradients.

In a black box-approach, we may look at the tube as a transfer element, characterized by an input x, an output y, and a transfer function y = τ(x). Because of the tube's rigidity and the resulting constancy of hydraulic conductance, the transfer function τ(x) is a linear function of the type y = b × x. Provided the system comprises only the said tube in the system with no further hydraulic conductance in series, then τ'(x) corresponds to the hydraulic conductivity L_A _of tube A.

We can add a second, "virtual" second output z = β(x) to the black box that expresses the flow in a putative second hydraulic resistance in series, induced by a corresponding fraction of the total pressure gradient. Here, there is no second resistance, and z trivially assumes a value of 0. We can thus formulate a buffered system as:



When computing the four buffering parameters t, b, T, and B, it becomes apparent that all four are again dimensionless, even though this buffered system is dimensionally heterogeneous. Under the indicated conditions, these parameters are



Buffering coefficient b and buffering ratio B both equal zero, in agreement with the absence of any buffering.

#### Linear buffering of flow against pressure changes

Next, we add a second piece of tubing. When connected *in parallel*, this tube B does not affect the pressure-flow relationship, only the relation between total flow and individual flow. In contrast, when the second piece of tubing is connected *in series*, the pressure-flow relation is altered profoundly. Analogously, autoregulation of blood flow in living organisms may be brought about via modulation of hydraulic conductance (by constriction or relaxation) of blood vessels that are in series with the capillary bed and that belong to the organ's proper vascular bed (i.e., they are located between the two points that used to define the relevant pressure gradient). Whether this resistance is located upstream, downstream, or both is not relevant for the pressure gradient, albeit these variations do affect the transmural pressure. A well-studied example is the autoregulation of glomerular blood flow via afferent and efferent arterioles of renal glomeruli.

Thus, this alternative definition equates "autoregulation" with the deviation of an observed hydraulic conductance from an expected "normal" or "standard" value (usually the intrinsic conductance of the isolated hydraulic conductor, e.g. the glomerular capillaries). Consequently, a lumped series resistance (e.g. pre- or postglomerular sphincters) can explain and replicate this type of autoregulation. Moreover, autoregulation in this sense originates in the organ itself (e.g. the kidney) and can therefore be studied in an isolated organ.

In quantitative terms, the addition of a second tube of identical dimensions, for instance, halves the volume flow at a given overall pressure difference. Put differently, the associated "apparent hydraulic permeability" Δφ_i_/ΔP of tube A is reduced to one half of its original value. Inasmuch as a given pressure change ΔP now results in a smaller change of volume flow as compared to the situation without series resistance, we can say that volume flow is now "buffered" against pressure changes. In terms of a buffered system, we represent this situation as



from which the buffering parameters follow immediately as



With an independent variable x having the dimension of a pressure and the corresponding two dependent variables y, z having the dimensions of a flow, the system is dimensionally heterogeneous, and this necessarily implies that it is also non-conservative or "distorted" (σ'(x) ≠ 1). The distortion is a linear one because σ'(x) = L_A _= constant *(Buffering I *14*)*.

Importantly, the buffering parameters can be computed only if the sigma function σ(x) is defined explicitly or implicitly. This function, the sum of τ(x) and β(x), specifies the response σ'(x) of the system in the absence of buffering where β'(x) = 0 and thus τ'(x) = σ'(x). In other words, one can talk meaningfully about buffering only if one is able to identify a reference state where buffering equals zero by definition, and to obtain a quantitative description of the system under these conditions. This step is crucial, but not necessarily trivial, particularly in dimensionally heterogeneous systems.

The sigma function provides the clue to the quantitation of buffering in more complicated situations where the unbuffered response itself is non-linear *(see paragraph on blood pressure variability buffering in *Additional file 4), or where the second output is a completely virtual, abstract quantity, such as in the context of systems and control theory (Additional file 6). Even when such a reference state exists, it may be inaccessible experimentally.

However, identification of a reference state may as well be impossible as a matter of principle, indicating that rigorous quantitation of buffering strength in this case is inherently impossible and the word "buffering" could then be used merely in a metaphorical way. One such example is blood pressure buffering in the sense of "blood pressure variability buffering"; Additional file 4 contains our criticism of this term.

#### Non-linear buffering at linear pressure-flow relationship

In a further modification of our model, we replace the second, rigid "buffering" tube by an elastic one, while retaining the first, rigid "transfer" tube with its constant hydraulic permeability L_A_. The pressure-flow relation becomes non-linear for both outputs y = τ(x) and z = β(x). Similarly, the buffering parameters t, b, T, and B become dependent on x and must be written as t(x), b(x), T(x), and B(x), respectively. Only in the complete absence of buffering, blood flow will be a linear function of perfusion pressure, implying that σ'(x) = constant.

Note that flow in an elastic tube depends not only on the pressure difference, but on the absolute pressure as well; therefore, it does make a difference whether the second tube is placed upstream or downstream to the first one. In principle, the serial arrangement of one rigid and one elastic tube with an appropriate pressure-conductance profile can reproduce all possible pressure-flow relationships. From a mechanistical point of view, however, the assumption that the unbuffered system should exhibit a perfectly linear pressure-flow relationship (modeled by a rigid tube) appears unrealistic.

#### Non-linear pressure-flow relationship – "distortion"

Unlike the rigid tube in the foregoing example, the blood vessels of most organs exhibit a highly non-linear relation between pressure difference and volume flow, even upon complete inhibition of vasomotion or other active regulation of hydraulic permeability. Here, non-linearity does not mean "buffering". Rather, it reflects solely the passive-elastic properties of the vessels as determined by vessel architecture and material; an appropriate single elastic tube may replicate such a non-linear relationship. One may therefore posit the pressure-flow relationship observed under these conditions as the unbuffered system response. Flow is now the product of pressure difference and a hydraulic conductance that varies non-linearly with absolute pressure: φ_1 _= ΔP × L(P).

In general terms, independent from hydraulic quantities, the sigma function σ(x) is then a non-linear function σ(x). This means that the system responds non-linearly to changes of the independent variable even in the absence of buffering; this behavior was termed above "non-linear distortion". If there exists anything like a "normal", "unbuffered" response with respect to organ perfusion in response to blood pressure changes, then it may be expressed exactly by such a sigma function.

Any modification of that normal response would then constitute "buffering"; in the present example, buffering can be brought about, for instance, by mechanisms such as contraction of smooth muscles in pre- or postcapillary sphincters. With an explicit specification of the unbuffered system response (in terms of the sigma function σ(x)), it is then straightforward to derive an explicit quantitative expression of the four buffering parameters from the observed buffered system response (given by the transfer function τ(x):



and



As in the preceding example, the coefficients b(x) and t(x) vary non-linearly with x.

Taken together, the formal and general concept of buffering not only allows one to quantitate buffering action in conservative systems, but can be applied with similar rigor to dimensionally heterogeneous systems and to systems with a non-linear response in the unbuffered state. When one wishes to compare different systems in terms of these measures of buffering action, it becomes necessary to recall the distinction between "normalization in x" vs. "normalization in y, z" *(Buffering I *14*)*: The extent of autoregulation in various organs can be compared either *at similar pressures *(either directly or upon "normalization in x" of the respective pressure-flow curves), or *at similar volume flows *(upon normalization in y, z). Both perspectives may make sense, and it is necessary to explicitly specify which one is used.

The concept of "non-conservative buffered systems" can be applied analogously to other phenomena. For instance, Additional file 4 applies this concept to electric phenomena, leading among others to a quantitative measure of rectification.

## Time-dependent buffering of cytoplasmic Ca^++ ^ions – The concept of "muffling"

### The basic concept of "muffling"

The buffering parameters t, b, T, or B all describe the relation between the derivatives of two functions of a common independent variable. Invoking the paradigm of "buffering" therefore requires that the phenomenon in question indeed exhibits a reproducible, well-defined relationship between three variables. Furthermore, one must be able to identify and express that relationship in an explicit mathematical form – namely, as an ordered combination of two functions or "buffered system". In practice, this usually means that the analysis is restricted to time-independent systems, or to the time-independent equilibrium states of a given time-dependent system. For instance, immediately following the addition of H^+ ^ions to a solution, ion concentrations will transiently change until a stable and characteristic end-point is reached with respect to the partitioning of total H^+ ^ions between water ("free") and other H^+ ^acceptors ("bound"). Acid-base chemistry is largely occupied with these stable end-points.

On the other hand, the presence of buffers not only determines the position of the final equilibrium, but also affects the path and the speed with which this equilibrium is attained. Often enough, the details of these pre-steady state events are practically relevant. For instance, the particular shape of the free Ca^++ ^concentration transients in response to acute Ca^++ ^loads can modulate cell signalling in neurons. Strong Ca^++ ^buffering may protect from cell death under certain pathological conditions [11], but may as well cause or aggravate cell damage in other situations [5]. Clearly, having a quantitative measure of buffering action during the pre-steady state is of similar scientific interest as having such a measure for steady-state buffering.

Obviously, the Ca^++ ^transients in live cells differ profoundly from those observed in pure water or saline. However, the Ca^++ ^transients also vary considerably from one cell type to another [5]. The two major mechanisms that shape such transients are binding (i.e., to ligands within the volume element under study) and transport (i.e., net flux across the boundaries of the volume element). Transport may be brought about by *i) *diffusion of free ions, *ii) *diffusion of ions bound to buffer molecules, and *iii) *channels or carriers that translocate free or bound ions across membranes.

The combined effect of binding and transport on the transients elicited by an acute ion load has been termed "muffling" [2]. In many experimental systems, muffling appears to be a rather complex process, and the observed time courses may follow complicated, multiphasic patterns. Is it possible under such circumstances to find a general quantitative measure of "muffling"? Ideally, such a measure should again provide a ratio scale and be as general and rigorous as the scale used to quantitate buffering strength under equilibrium conditions.

### A ratio scale for muffling

#### The response to a concentration step in a small volume element

**Figure 3:**
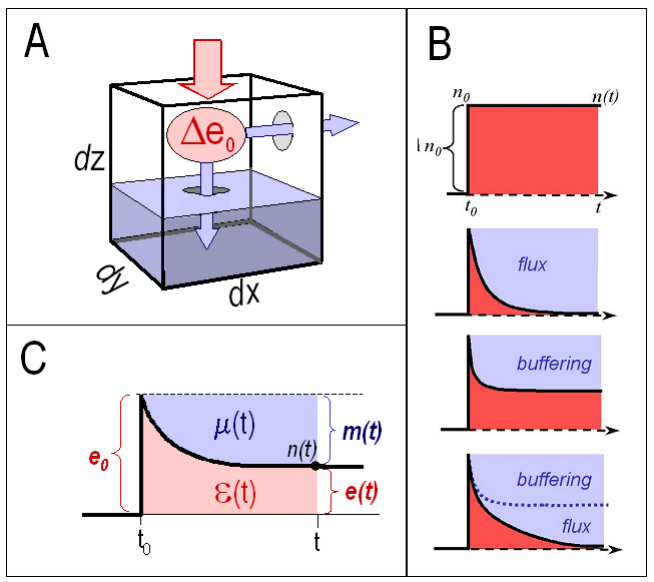
***Time-dependent buffering: the "muffling" of Ca^++ ^ions***. ***A, Muffling is brought about by binding and flux***. The concentration of free Ca^++ ^ions in a small volume element *(dx × dy × dz) *is changed instantaneously by addition Δe_0 _further free Ca^++ ^ions *(red arrow)*. The "error" imposed by this acute Ca^++ ^load either persists, or it is reduced over time. Reduction *(blue arrows) *may occur via Ca^++ ^flux across the boundaries of the volume element *(right arrow)*, or by binding of Ca^++ ^to Ca^++ ^buffers within the volume element *(lower arrow); *the time-dependent, combined effect of binding and flux is termed "muffling". ***B, Prototypes of muffling***. From top to bottom: 1, Zero muffling in the absence of both flux and binding; 2, Muffling via flux across its boundaries (e.g. diffusion), without binding to buffers within the volume element; 3, Muffling via binding to buffers inside the volume element, without flux; 4, Muffling due to both binding and flux. Note that peak size and magnitude of muffling action are not correlated. ***C, Measures for time-dependent "error reduction", or "muffling"***. A measure that reflects both the size and the duration of the error e(t) is the integral *(red area)*. Similarly, a measure that reflects both the size and the duration of the "error reduction" or "muffling" m(t) is given by the integral *(blue area)*. The proportions between ε(t) and μ(t) can be used to define a "muffling coefficient" and "muffling ratio" *(see main text of BufferingII)*. These measures are the time-dependent analogs of the time-independent "buffering coefficient" and "buffering ratio" *(Buffering I)*.

We consider a volume element Δx × Δy × Δz assumed to be homogenous with respect to the relevant ion concentrations (Figure 3A). Let this volume element contain a solvent and a specific solute, with some solute molecules being bound, others "free". The initial number of "free" molecules is denoted n_0_.

Now, the system is acutely disturbed by adding (or removing) instantaneously a certain amount e_0 _of the free molecules. Then, the number of free molecules will instantaneously jump from its baseline value n_0 _to a value n(t) = n_0_+e_0. _If present, "muffling" according to the above definition (i.e., binding to buffer molecules within the volume element, or net flux across its boundaries) will tend to return n(t) towards n_0 _over time and thus decrease the absolute deviation |e(t)| (these considerations are valid for addition as well as for removal of solute, and both e_0 _and e(t) may thus have a positive or a negative sign). Depending on the relative contributions of binding vs. flux, three limiting cases can be distinguished (Figure 3B).

##### A. Zero muffling (Figure 3B, top)

If there is neither binding nor net flux, the initial disturbance e_0 _will persist, and e(t) will remain constant at e(t) = e_0_. A measure that reflects both size and duration of the deviation e(t) is the integral of e(t) over time, which we term ε (Figure 3C):

.

In this case, ε(t) will increase linearly over time. The letters e and ε may be associated with "error" or "extra ions".

##### B. Muffling via flux (Figure 3B, 2^nd ^from top)

If the solute exhibits net flux into adjacent volume elements, but no binding within the volume element itself, then some of the added solute molecules e_0 _will leave the volume element and thus decrease e(t). Herein, the final equilibrium concentration of the solute depends on size and composition of the adjacent volume elements, and on the available active transport mechanisms. For instance, most cell types will sooner or later extrude a cytoplasmic Ca^++ ^load completely, with e(t) approaching zero over time. With efflux, the error integral ε(t) increases more slowly over time than without.

The error e(t) = n(t)-n_0 _is but one way of looking at such a muffling process. Alternatively, one can measure the extent by which the initial error e_0 _is reduced over time; this "error reduction" is equal to the difference m(t) ≡ e_0_-e(t). Again, size and duration combined of this error reduction m(t) are faithfully reflected by an integral, i.e., the integral of m(t) over time, which we term μ (Figure 3C):



The letters m and μ may be associated with "muffling".

##### C. Muffling via binding (Figure 3B, 3^rd ^from top)

In the absence of net flux (due to the lack of appropriate gradients or permeabilities), but with binding of solute within the volume element itself, the deviation e(t) of solute concentration from its initial value n_0 _will be reduced to some equilibrium value between 0 and e_0_. Again, the situation can be described in terms of the error e(t), error reduction m(t), and their respective time-integrals ε(t) and μ(t).

#### Definition of the "muffling ratio" M(t)

These examples show that the magnitude of muffling depends on several aspects, including not only the size of the initial "peak" e_0_, but also the speed by which that disturbance is counteracted and the steady-state error e(t) that remains during the final equilibrium. As a quantitative measure M(t) of muffling at a given time, we can use the ratio of the error reduction integral μ(t) over the error integral ε(t) (Figure 3C):



This measure is formed analogously to the buffering ratio B. We term it "muffling ratio". From a chemically more realistic perspective, the states of the solute are statistical averages. The muffling ratio can thus be interpreted as the ratio of the average number  of added molecules that existed in a muffled state (i.e., bound or outside the volume element under study) within the particular time window, over the average number  of added molecules found free and inside the volume element during that time:



#### Properties of the muffling ratio M(t)

M(t) always is a dimensionless number, and yields an absolute ratio scale for muffling strength. If an error e_0 _persists fully, the muffling ratio assumes a value of zero. An error e_0 _that is counteracted completely and instantaneously will yield infinite muffling ratio. Importantly, the specific value of M(t) in a given system usually depends on multiple conditions and parameters (initial solute concentration, magnitude and direction of the initial disturbance, integration time), with no *a priori *constraints on any of them. In order to be completely unambiguous, the relevant parameters should be indicated along with the muffling ratio in the form M(n_0_, e_0_, t). In other words, it is not possible to characterize muffling by a single characteristic parameter.

In most cases, buffering and transport tend to *decrease *the absolute error |e(t)| over time. Then, the muffling ratio M(t) always has a positive sign, even when ε(t) and μ(t) are both negative. On the other hand, meaningful negative values of M(t) can be obtained under certain conditions: For instance, muffling mechanisms may cause the absolute error |e(t)| to increase further beyond |e_0_|, or they may produce an "undershoot" or "overshoot". Finally, the instantaneous disturbance e_0 _may elicit oscillations of n(t), either dampened or undampened ones. The value of M(t) as a function of time will then oscillate, too, and may include negative values.

#### Buffering is a limiting case of muffling

Importantly, our two concepts of buffering and muffling are linked conceptually and numerically. Recall that "buffering" refers to the proportion between the changes of two time-independent partitioning functions τ(x) and β(x) in response to an infinitesimal perturbation, and "muffling" to the proportion between two time-dependent functions ε(t) and μ(t) in response to a finite perturbation e_0_. Some (not necessarily all) muffled systems will travel over time towards a unique, time-independent equilibrium upon a particular disturbance e_0_.

As demonstrated amply above, such time-independent equilibrium states can be described in terms of time-independent transfer and buffering functions, τ(x) and β(x), and therefore also by the four buffering parameters t, b, T, and B. The initial equilibrium state is given by {τ(x_0_), β(x_0_)}, and the equilibrium after imposing the disturbance e_0 _by {τ(x_0_+e_0_), β(x_0_+e_0_)}. Comparing the equilibrium states before and after a finite disturbance e_0_, the transfer function τ(x) changes by an amount

Δτ = τ(x_0_+e_0_) - τ(x_0_),

and the buffering function β(x) by an amount

Δβ = β(x_0_+e_0_) - β(x_0_).

Herein, the two partial changes equal the initial disturbance, in keeping with the conservation condition: Δτ+Δβ = Δx = e_0_.

The limit of the error integral ε(t) for infinitely long integration time is t × Δτ, and the corresponding limit of the error reduction integral μ(t) is equal to t × Δβ. Thus, the muffling ratio at infinite time is



The ratio Δβ/Δτ reflects the average buffering ratio  in the interval between x_0 _and x_0_+Δx, but is different from the true buffering ratio B = dβ/dτ at x = x_0_.

In a second limit process, we let the perturbation e_0 _decrease progressively from a finite value towards zero. The ratio  will then approach the differential , i.e., the buffering ratio B(x):



Thus, for long integration times and small perturbations e_0_, the muffling ratio becomes equal to the buffering ratio. In other words, buffering is a special limiting case of muffling for t→∞ and e_0_→0.

Indeed, these limiting conditions are fundamental in any attempt to determine time-independent buffering power rather than time-dependent muffling, whatever particular buffering unit is to be employed. For instance, determination of H^+ ^buffering power from the slope of the titration curve [12] requires that the solution be stirred well and for long enough to allow for complete equilibration (t→∞), and to use small amounts of titrant (e_0_→0).

Note that "muffling" according to our definition provides an empirical measure extracted from concentration transients, but it does not imply any mechanistical assumptions, such as the distinction between binding and transport processes. Inasmuch as buffering is a limiting case of muffling, buffering may similarly comprise both binding and transport. Time-dependent and -independent responses to disturbances are shaped by the combined action of these two mechanisms, and it therefore seems appropriate that the units for buffering and muffling reflect these combined effects. Restricting buffering or muffling by definition to its binding component would leave us without a measure for the effects caused by net transport, and complicate experimental approaches without increasing biological validity. – The properties of our muffling strength unit are discussed further and compared to previous approaches in the Additional file 5.

#### Muffling can be viewed as "dynamic disturbance rejection"

In principle, muffling of a Ca^++ ^constitutes a specific form of "dynamic disturbance rejection", which is one aspect of "control". Analogously, the effect of H^+ ^buffers on steady-state pH can be viewed as "static disturbance rejection". It is straightforward to extend the buffering concept in order to obtain measures of "systems level buffering strength". Specifically, these measures allow one to quantitate the efficiency of "setpoint tracking" and of "disturbance rejection" by control systems, either static or dynamic. These measures have been sketched elsewhere [13]; a more systematic and comprehensive outline is found in Additional file 6. Formulating the buffering concept in the language of systems and control theory conveys a tangible quantitative meaning to the term "resistance to change", the customary paraphrase of "buffering".

## Conclusion

*When you can measure what you are talking about and express it in numbers, you know something about it*. -Lord Kelvin

In this article, we applied the formal and general concept of buffering presented in the preceding paper *(Buffering I *14*) *to various types of buffering phenomena. The buffering of H^+ ^ions in solutions of a weak acid and in pure water could be analyzed simply in terms of "conservative buffered systems". The buffering of organ perfusion in the face of variable blood pressure made it necessary to invoke the concept of "non-conservative", "dimensionally heterogeneous" systems. Describing the response of a cell to an acute Ca^++ ^ion load could be achieved with a novel quantitative measure of the time-dependent aspects of buffering, also termed "muffling". Muffling is equivalent to what is called "dynamic disturbance rejection" in systems and control theory, and our general concept of buffering yielded further quantitative measures of control performance. These measures allow one to describe all major aspects of control, namely static or dynamic ones, and disturbance rejection as well as setpoint tracking.

### Most buffering phenomena may be interpreted in terms of the control paradigm

Control systems may exhibit complex buffering behavior, and their description then requires all four measures of control efficiency. Conversly, the control paradigm provides, in the form of these measures of control efficiency, a sufficient framework for the quantitation of *all *buffering phenomena, including phenomena that are not routinely viewed in terms of control systems. For instance, the addition of H^+ ^ions to an aqueous solution can be interpreted as "disturbance", and the "buffering" of H^+ ^ions as "static disturbance rejection". Both "buffering" and "static disturbance rejection" can be quantitated in terms of the buffering ratio B(x), which implies that both are just different words for the same thing. Similarly, the addition of Ca^++ ^ions to the cytoplasm may be interpreted as a "disturbance", and the extent to which this Ca^++ ^load is counteracted in a given time window is reflected in the "dynamic disturbance rejection ratio" M(t).

It is a very common finding that buffering phenomena in a biological context serve apparent homeostatic or control purposes. Then, "buffering strength" is directly proportional to "control efficiency". In fact, they are the same thing, and our concept of buffering provides the corresponding unifying formal framework. In contrast, it is impossible to establish any systematic connection between Van Slyke's buffering strength unit (which is expressed in terms of "moles per liter" [12]) and any known measure of control efficiency.

### The abstract definition of the buffering concept is compatible with multiple, different interpretations

In many cases, the systems and control paradigm thus provides an intuitive and fruitful interpretation of the static and dynamic buffering parameters. For instance, such an interpretation is widely applicable in the areas of physiology and systems biology. Nonetheless, one should maintain the distinction between the original abstract definitions (stated in *Buffering1 – main text *14 and in axiomatic form in *Buffering1 *14– Additional file 7) and their subsequent interpretations. "Control" is but one out of several possible interpretations of these measures. "Probability" would be another natural interpretation, given that these measures were based in our axiomatic approach on a "signed probability measure" and were formulated as a "non-Kolmogorov axiomatic systems of probability" *(Buffering I *14– Additional file 7). The ambiguous relation between axioms and their interpretations is a general finding; completely analogous to the buffering parameters discussed here, Kolmogorov's probability axioms have multiple interpretations, e.g. as relative frequencies, geometric probabilities, degree of individual belief, propensities etc The axioms are logically consistent, but their various interpretations may be (and usually are) in mutual logical conflict. Moreover, no single interpretation allows comprehensive treatment of all phenomena encountered by researchers.

Maintaining the distinction between axioms and interpretation of axioms prevents spurious conflicts between interpretations, and allows one to use the axioms in a given situation flexibly and in the most appropriate way. In the words of Bertrand Russel: "It must be understood that there is here no question of truth or falsehood. Any concept which satisfies the axioms may be taken to *be *mathematical probability. In fact, it might be desirable to adopt one interpretation in one context, and another in another." *("Human Knowledge", 1948)*

Taken together, we have established a formal and general concept for the quantitation of buffering action, demonstrated the practical usefulness of that concept in a variety of contexts, and provided explicit descriptions of several important buffering phenomena. The formal and general concept is of theoretical interest with its underlying "non-Kolmogorov" axiomatic system of probability that is based on a non-Boolean bag algebra and accomodates negative as well as variable probabilities. On the practical level, our concept solves the problem of quantitating buffering action, and provides a common scientific language for a common quantitative pattern that is present in very diverse phenomena, and in many disciplines.

## Supplementary Material

Additional File 2 H^+ ^Buffering in Pure Water Click here for file

Additional File 3 Other Conservative Buffered Systems Click here for file

Additional File 5 Notes on Time-Dependent Buffering or "Muffling" Click here for file

Additional File 6 Buffering and Muffling in Systems and Control Theory Click here for file

Additional File 1 Further Notes on Langmuir Buffering Click here for file

Additional File 4 Other Non-Conservative Buffered Systems Click here for file
