# Supplementary material for: The quantitation of buffering action II. Applications of the formal & general approach
Source: Theor Biol Med Model. 2005 Mar 16;2:9. doi: 10.1186/1742-4682-2-9 (PMC1079954; doi:10.1186/1742-4682-2-9)
Supplement: Additional File 2 — H+ Buffering in Pure Water [file 1742-4682-2-9-S2.pdf]

Theoretical Biology and Medical Modelling

Research

The quantitation of buffering action. II. *Applications of the formal and general approach.*

Bernhard M. Schmitt

Supplement 2:

H^+ Buffering in Pure Water

Buffering of H^+ and OH^- ions in pure water probably represent the most primal physico-chemical buffering phenomena, and their quantitative description is of great interest. In this context, "pure water" shall mean water without any solutes that could act as H^+ buffers. In the following, it is understood that addition of H^+ ions to pure water, or removal of H^+ ions from it is carried out in the form of "strong" acids or bases, respectively.

To obtain an explicit quantitative description of buffering in pure water according to our concept of buffering *(Buffering I)*, we first recapitulate a standard mathematical model describing the concentrations of OH^- and H^+ ions in water as functions of added strong acid or base. Then, we form the corresponding "buffered system" and derive from it the four buffering parameters t, b, T, and B.

Quantitative description of $[OH^-]$ and $[H^+]$ concentrations in pure water with added strong acid or base

Water molecules dissociate into H^+ and OH^- ions which are said to be "free" in solution; the actual chemical details are more complex and subject to debate, but not relevant to our argumentation. The extent of dissociation in pure water is given by a constant K_W that lumps together water concentration and the dissociation constant of water:

$$K_W = K_d \times [H_2O] = [OH^-]_{free} \times [H^+]_{free}$$

Herein, all terms are concentrations and hence positive-valued. The term $[H_2O]$ can be treated as a constant under most conditions because the concentration of water is ~55.5 M and thus many orders of magnitude higher than its K_d. Similarly, K_W is a well-known constant (10^{-14} M^2 at 22°C; for other

temperatures, note the strong increase of K_W with temperature).

Addition of strong acid or strong base will change total H^+ ion concentration by a certain amount $\Delta[H^+]_{total}$. The concentration of free H^+ ions will change in the same direction, but to a lesser extent and in a way that is neither linear nor immediately evident. However, the following equation will always hold:

$$[H^+]_{free} - [OH^-]_{free} = \Delta[H^+]_{total}$$

When strong acid is added, $[\Delta H^+]_{total}$ has a positive sign, and a negative one when strong base is added. With two equations and two unknowns ($\Delta[H^+]_{total}$ and $[H^+]_{free}$), the relationship between the unknowns is determined completely. We thus obtain the mathematical representation of H^+ ion concentration in water with added or removed acid as:

$$[H^+]_{free} = \frac{\Delta[H^+]_{total}}{2} + \sqrt{\left(\frac{\Delta[H^+]_{total}}{2}\right)^2 + K_W} \; .$$

The dependence of $[OH^-]_{free}$ on $\Delta[H^+]_{total}$ can be calculated analogously. The quantitative relation between $[H^+]_{free}$, $[OH^-]_{free}$, and $\Delta[H^+]_{total}$ is summed up graphically in *Figure 1* of this Supplement. This mathematical model of water ignores issues such as ion activity vs. ion concentration, or the deviation of $[H_2O]$ from 55.5 M at very high values of $[OH^-]_{free}$ or $[H^+]_{free}$. Within wide limits, however, this equation is sufficiently close to chemical reality to be meaningful. Importantly, previous analyses of H^+ buffering in pure water were made on the basis of the same model [1-4], and adhering to it will allow us to compare directly the conclusions obtained with the various approaches.

Water as a "buffered system"

Next, we turn this description of elementary physico-chemical events into a "buffered system", i.e., an ordered pair of functions of one common variable. As "transfer function" $\tau(x)$, we choose free H^+ concentration $[H^+]_{free}$ as a function of $\Delta[H^+]_{total}$, and rewrite it in a more general notation:

$$\tau(x) = y = \frac{x}{2} + \sqrt{\left(\frac{x}{2}\right)^2 + 1}$$

For clarity and simplicity, all concentration terms herein are given as multiples of $\sqrt{K_W}$, i.e.,

$$x \Leftrightarrow \Delta[H^+]_{total} / \sqrt{K_W}$$

and

$$y \Leftrightarrow [H^+]_{free} / \sqrt{K_W} \; .$$

This form reduces computations to operations with dimensionless numbers and is generally valid, independent from the particular value of $\sqrt{K_W}$ which depends on temperature, pressure and other variables. Because H^+ ions are neither destroyed nor created in the process of buffering, the buffered system must be a conservative one, i.e., $\tau(x)+\beta(x)=x+c$. From the transfer function $\tau(x)$ and the conservation condition, the buffering function $\beta(x)$ follows as:

$$\beta(x) = z = c + \frac{x}{2} - \sqrt{\left(\frac{x}{2}\right)^2 + 1} \qquad .$$

We know that the buffering parameters **t**, **b**, **T** and **B** as differentials do not depend on the constant **c**, and so we may set **c=0** for simplicity. Thus, the buffered system constituted by this mathematical model of water, denoted B_{water}, has the form of the following ordered pair of functions:

$$B_{water} = \{[H^+]_{free} \, ; [H^+]_{bound}\} = \{\tau(x); \beta(x)\}$$

$$= \left\{ \frac{x}{2} + \sqrt{\left(\frac{x}{2}\right)^2 + 1} \; ; \; \frac{x}{2} - \sqrt{\left(\frac{x}{2}\right)^2 + 1} \right\} \; .$$

Herein, the independent variable x and the functions $\tau(x)$ and $\beta(x)$ are all dimensionless numbers: The system is "dimensionally homogeneous". Without the simplification of dividing all concentration terms by $\sqrt{K_W}$, all three were of the same dimension of "moles per liter", again constituting a dimensionally homogeneous system.

Computing the buffering parameters t, b, T, and B

In a conservative system with $\sigma'(x)=1$, transfer coefficient $t=\tau'/\sigma'$ and buffering coefficient $b=\beta'/\sigma'$

simply correspond to the derivatives of transfer function τ and buffering function β:

$$t(x) = y'(x) = \tau'(x) = \frac{1}{2} + \frac{x}{4 \times \sqrt{\frac{x^2}{4} + 1}}$$

$$b(x) = z'(x) = \beta'(x) = \frac{1}{2} - \frac{x}{4 \times \sqrt{\frac{x^2}{4} + 1}}$$

From **t** and **b**, we compute the transfer and buffering ratios as

$$T(x) = \frac{t(x)}{b(x)} = \frac{\sqrt{x^2 + 4} + x}{\sqrt{x^2 + 4} - x}$$

$$B(x) = \frac{b(x)}{t(x)} = \frac{\sqrt{x^2 + 4} - x}{\sqrt{x^2 + 4} + x}.$$

The latter two equations become considerably simpler when the parameters are expressed as functions of **y** or **z**:

$$T(y) = \frac{1}{z^2} = y^2$$

and

$$B(y) = \frac{1}{y^2} = z^2.$$

In terms of the known constant K_W and the directly measurable pH (or $[H^+]_{free}$), one can express **T** and **B** as

$$T = \left(\frac{[H^+]_{free}}{\sqrt{K_W}} \right)^2$$

and

$$B = \left(\frac{\sqrt{K_W}}{[H^+]_{free}} \right)^2.$$

These parameters give the complete and quantitative description of H^+ buffering in pure water *(Figure 1B)*.

Figure 1: The buffering of H^+ ions in pure water.

A, Concentrations of free H^+ ions, free OH^- ions, and added strong acid in pure water.

All concentrations are expressed as dimensionless multiples of $\sqrt{K_W}$, where K_W is the ion product of pure water. Negative values on the axis representing $\Delta[H^+]_{total}$ correspond to the addition of strong base. *Black curve:* the relation between the three variables, based on the assumptions that the ion product of water is constant and that added protons are conserved. *Green circle,* neutral point, where $[H^+]=[OH^-]$. *Filled curves:* Projections of the thick curve, corresponding to the individual relations between $\Delta[H^+]_{total}$ and $[H^+]$ *(red)* or $[OH^-]$ *(blue)*. Note the absence of maxima or minima, and of symmetry around any of the axes.

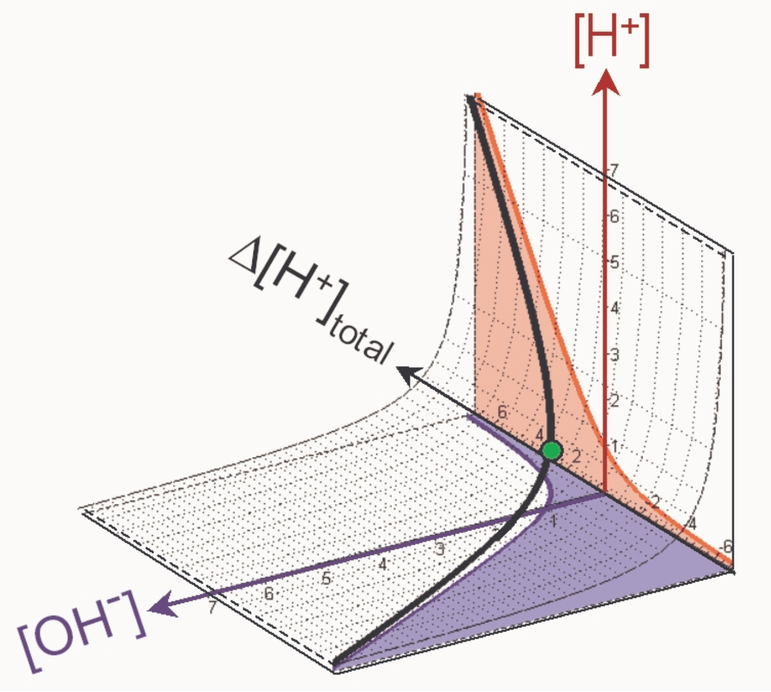

Figure 1 (ctd.)

B, Describing H^+ buffering in pure water using the four buffering parameters t, b, T, and B.

Titration of pure water to more acidic and more alkaline values. 0 on the y-axis indicates the neutral point. Concentrations of added strong acid or strong base *(x- axis)* and of additional free H^+ ions *(y-axis in top row)* expressed as dimensionless multiples of $\sqrt{K_W}$.

Top row: Left panel: τ*,* the change in free H^+ ion concentration as a function of the change $\Delta[H^+]_{total}$ of total H^+ ion concentration; this function is termed "transfer function τ". *Right panel:* β*,* "buffering function β", i.e., the difference between added total H^+ ions and additional free H^+ ions as function of the change in total H^+ concentration. This difference represents the H^+ ions that were "buffered".

Middle panel, left: "Transfer coefficient t", i.e., the (differential) fraction of added H^+ ions that partition into the pool of free H^+ ions at a given state of the system. *Middle panel, right:* "buffering coefficient b", i.e., the (differential) fraction of added H^+ ions that do *not* partition into the pool of free H^+ ions.

Bottom panel, left: "Transfer ratio T", i.e., the (differential) ratio of additional free over additional buffered H^+ ions. *Bottom panel, right:* "Buffering ratio B", i.e., the (differential) ratio of additional buffered over additional free H^+ ions (the reciprocal of the transfer ratio).

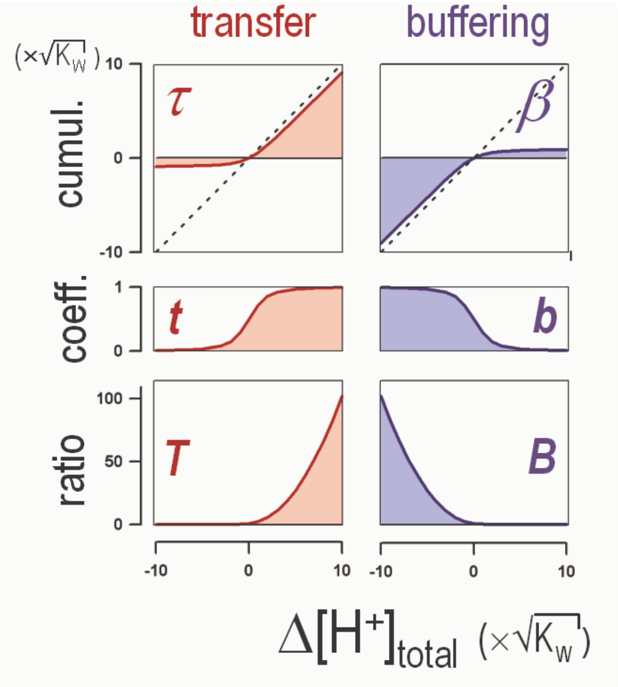

C, H^+ buffering in pure water according to the "buffer value" β_{H^+}

(Michaelis, 1920, and Van Slyke, 1920).

Top panel: Titration of pure water as in *Figure 1B,* except that the concentration of free H^+ ions *(y-axis)* is not plotted on a linear scale, but was transformed logarithmically into pH values according to the relation $pH = -\log_{10}\left(\frac{[H+]\times liter}{mole}\right)$. In such a plot, the slope of this titration curve decreases symmetrically on either side of the neutral point.

Bottom panel: Buffer value β_{H^+} as defined by Michaelis and Van Slyke: $\beta_{H^+} = d[Base]/dpH = -d[H^+]_{total}/dpH$. This buffer value is equivalent to the inverse of the absolute value of the slope of the titration curve shown in the top panel. Using this definition of buffering strength, the buffering process now appears biphasic and symmetrical due to the logarithmic transform (cf. the monophasic, asymmetrical behavior observed in *Figure 1B, bottom panel*).

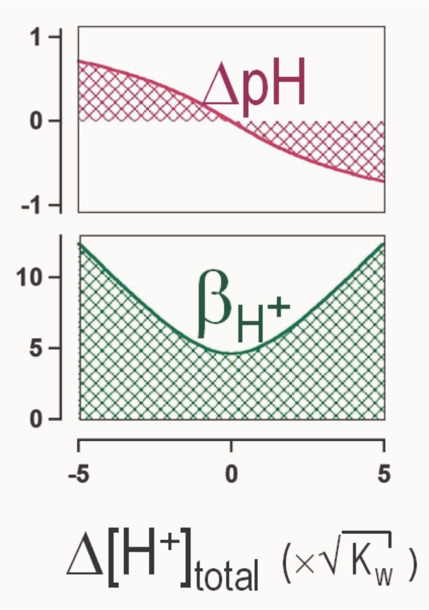

Characteristics of H^+ buffering in water as described by the parameters t, b, T, and B

For H^+ buffering in pure water, transfer and buffering function have direct chemical meanings.

The value of the transfer function $\tau(x)$ represents free H^+ ion concentration. The buffering function is always negative-valued. Interestingly, its absolute value equals the hydroxyl ion concentration:

$$\beta(x) = -[OH^-]_{free} \ .$$

This is a consequence of our model assumption $[H^+]_{free} - [OH^-]_{free} = \Delta[H^+]_{total}$, which can be rearranged to

$$-[OH^-]_{free} = \Delta[H^+]_{total} - [H^+]_{free} \Leftrightarrow x{-}y{=}z{=}\ \beta(x).$$

With respect to H^+ ions, pure water is a non-linear, non-inverting moderator with infinite buffering capacity.

The slope of the transfer function $\tau(x)$, here equal to the transfer coefficient $t(x)$, approaches 1 as x increases towards $+\infty$, and approaches 0 as x decreases towards $-\infty$. Thus, H^+ buffering in pure water can be formally classified as moderation ($|t|<1$) that is non-inverting ($t\geq0$) and non-linear ($t,b \neq$ const.). According to the presented mathematical model, one can remove unlimited amounts of H^+ ions from water, and the buffering function β decreases without lower bound. For this reason, H^+ buffering in water exhibits "infinite capacity". This clear violation of a conservation law is due to limitations of the mathematical model, and affects the validity of any buffering parameter one derives from it.

Water has an K_W-independent "equipartitioning point" for H^+ ions, located at the neutral point.

The neutral point is characterized by the equality $[OH^-]_{free} = [H^+]_{free} = \sqrt{K_W}$ and $\Delta[H^+]_{total}=0$. Here, we find: $(\Delta[H^+]_{total} = 0) \Rightarrow (t=b=0.5) \wedge (T=B=1)$. Thus, b and B have fixed values at the neutral point, irrespective of the value of K_W. This implies that temperature, pH, or pOH *per se* do not affect buffering at the neutral point. Furthermore, equal values for buffering parameters vs. transfer parameters ($t=b=0.5$ and $T=B=1$) mean that, at the neutral point, half of the added H^+ ions bind to water (forming "free" H^+ ions) and half of them bind to OH^- ions (forming "bound" or "buffered" H^+ ions). Such a behavior is termed here "equipartitioning".

Outside the neutral range, H^+ buffering in pure water approximates either "perfect buffering" or "zero-buffering".

In practical terms, transfer and buffering coefficients stay close to 0.5 only in a small interval around the neutral point. Towards more negative or more positive values of $\Delta[H^+]_{total}$, these parameters quickly and very closely approach either 0 or 1, respectively. Outside the neutral range, pure water thus displays one of two extreme H^+ buffering behaviors: either virtually "perfect" H^+ buffering under alkaline conditions, or virtually "zero" H^+ buffering under acidic conditions. In this respect, the H^+ buffering behavior of water is highly asymmetric.

Generation of additional free H^+ ions predominates in the acidic range, consumption of OH^- ions in the alkaline range.

The number of H^+ ions added into pure water is conserved, irrespective of whether these ions remain free or become bound. Accordingly, the sum of the derivatives of transfer and buffering function equals unity. Thus, an added H^+ ion either generates a "free" H^+ ion, thus increasing the value of $\tau(x)$, or it consumes one OH^- molecule by combining with it into H_2O, thus decreasing OH^- ion concentration and making the value of $\beta(x)$ more positive. Evidently, only the second of these two processes – binding of H^+ ions to OH^- ions - constitutes "buffering" of H^+ ions. There are two corollaries to this:

The more acidic the solution is, the smaller its H^+ buffering power.

When $\Delta[H^+]_{total}$ increases, H^+ buffering decreases.

The more alkaline the solution is, the greater its H^+ buffering power.

When $\Delta[H^+]_{total}$ decreases, H^+ buffering increases.

OH^- buffering in water

By convention, a solution´s acid-base status is usually reported in terms of H^+ ions. Similarly, the instruments used in this context are called "pH meters" rather than "pOH meters", although the

two ion species are completely interdependent in aqueous solutions. In principle, all aspects of aqueous acid-base chemistry might be viewed and quantitated with equal right and stringency in terms of OH^- ions. However, this correspondance does not imply that "H^+ buffering" and "OH^- buffering" should be one and the same thing.

To obtain the explicit quantitative description of OH^- buffering in water, we first solve the above two basic equations for $[OH^-]_{free}$ rather than for $[H^+]_{free}$. This yields free OH^- ion concentration as a function of added strong base $\Delta[OH^-]_{total}$:

$$[OH^-]_{free} = \frac{\Delta[OH^-]_{total}}{2} + \sqrt{\left(\frac{\Delta[OH^-]_{total}}{2}\right)^2 + K_W} \ .$$

Analogous to H^+ buffering, we express again all concentration terms as dimensionless multiples of $\sqrt{K_W}$ and set this relationship as the transfer function $\tau(x)$, which yields:

$$\tau(x) = y = \frac{x}{2} + \sqrt{\left(\frac{x}{2}\right)^2 + 1} \, .$$

Formally, this equation and the buffered system that follows from it are identical to the one obtained in the analysis of H^+ buffering. Dependent and independent variables, however, are now OH^- ion concentrations, rather than H^+ concentrations. In the context of OH^- buffering, the transfer function therefore describes the generation of additional free OH^- ions in response to the addition of OH^- ions, and the buffering function the associated consumption of H^+ ions. Thus, the position of "alkaline" vs. "acidic" states is reversed with respect to the ordinate. As a consequence, OH^- buffering in water is different from H^+ buffering, but symmetrical to it with respect to the neutral point.

Characteristics of OH^- buffering in water as described by the four buffering parameters t, b, T, and B

OH^- buffering in pure water exhibits characteristics that are either identical to the characteristics of H^+ buffering outlined above, or complementary to it. We can therefore simply state these properties here, without further explanations.

i) With respect to OH^- ions, pure water is a non-linear, non-inverting moderator with infinite buffering capacity.

ii) Water has an K_W-independent "equipartitioning point" for OH^- ions at the neutral point. At the neutral point where $\Delta[OH^-]_{total}=0$, the buffering coefficient **b** and buffering ratio **B** for OH^- have fixed values:

$$\Delta[OH^-]_{total}=0 \Rightarrow t=b=0.5 \wedge T=B=1 \ .$$

These values are independent from K_W, and thus from pH, pOH, and temperature.

iii) When strong base is added to water, generation of OH^- ions predominates at alkaline values, consumption fo H^+ ions at acidic values, which implies the following two statements:

iv) The more *alkaline* the solution is, the smaller the OH^- buffering power. With increasing $\Delta[OH^-]_{total}$, OH^- ion buffering decreases.

v) The more *acidic* the solution is, the greater the OH^- buffering power. With decreasing $\Delta[OH^-]_{total}$, OH^- ion buffering increases.

vi) With the exception of the zone around the neutral point, pure water displays one of two extreme OH^- buffering behaviors: either virtually "perfect OH^- buffering" under *acidic* conditions, or virtually "zero-OH^- -buffering" under *alkaline* conditions. Thus, the OH^--buffering behavior of water is highly asymmetric.

Common features of H^+ and OH- buffering in water

Taken together, acid-base buffering in pure water is skewed in two respects: Firstly, each ion species individually (OH^- or H^+ ions) is buffered very well on one side of the neutral point, but very poorly on the other. Secondly, the positions of strong vs. weak buffering with respect to acidic and alkaline ranges are reversed for one ion species as compared to the other.

On the other hand, buffering behavior at the neutral point is surprisingly constant: not only is its magnitude identical for OH^- and for H^+ ions, but also independent from K_W, and thus independent from temperature, pH and pOH that prevail at the neutral point. Away from the neutral point on any

side, however, changes of K_W do affect buffering of OH^- and of H^+ ions. Specifically, a greater K_W is associated with smaller changes of buffering power.

Moreover, it is a remarkably simple and symmetrical result that transfer and buffering are of equal magnitude at the neutral point, with a value of 1 for B and T.

Communicating-vessels model of H^+ buffering in pure water

The buffering behavior of water with respect to H^+ ions can be visualized using the physical model of two communicating vessels *(Buffering I)*. In order to directly visualize the buffering ratio B, for instance, a cylindrical "transfer vessel" with a cross-sectional area of $A_{transfer}=1$ cm^2 may be connected to an appropriately shaped "buffering vessel". Appropriate shapes can be derived from the relation $B=1/y^2$ as $A_{buffer}=A_{transfer}/h^2$, where h is the height of the transfer vessel, and A_{buffer} the cross-sectional area of the buffering vessel.

At the neutral point, the cross-sectional area A_B of the buffering vessel would then be 1 cm^2, equal to the area $A_{transfer}$ of the transfer vessel. Towards more alkaline values, the area A_{buffer} increases strongly. At pH values of 10 and 13, for instance, the cross-sectional area of the buffering vessel would be equal to squares with edges of 10 m and 10 km length, respectively (assuming a temperature of 22°C). In the acidic range, e.g. at pH 4 and pH 1, the edges of the buffering vessel would measure a mere 10 μm and 10 nm, respectively.

The buffering of free OH^- ion concentration in response to added or removed OH^- ions is illustrated by the same model with the sole difference that the fluid and the scale bars now represent the concentration of OH^- ions rather than of H^+ ions.

Comparison with other buffering strength units.

Van Slyke's buffering value β_{H+}= dBase/dpH

Van Slyke used the same mathematical model of water as presented here, but a different measure of buffering power [2]. Use of his unit β_H+ = dBase/dpH results in a description of "self-buffering" in water that differs fundamentally, not just numerically *(Figure 1C)*:

i) β_H+ has an absolute minimum at the neutral point, whereas B does not have a minimum or maximum.

ii) At the neutral point, β_H+ depends strongly on K_W, whereas B invariably equals 1.

iii) When moving away from the neutral point, the value of β_H+ increases symmetrically to infinity on both the acidic and alkaline sides. In contrast, the pH dependence of B is strongly asymmetric: in terms of the vessel model, the vessel cross-sectional area quickly approaches electron microscopic dimension on the acidic side, and geographic survey dimensions on the alkaline side;

iv) According to Van Slyke, OH^--buffering power β_{OH^-}=dAcid/dpOH equals β_H+ exactly over the entire pH range, whereas our parameter B indicated opposite and monotonical pH dependence of H^+ and OH^- buffering strengths, arranged in mirror-image like fashion around the neutral point.

In all four aspects, it is the unit β_H+ which fails to reflect H^+ buffering faithfully. Common sense as well as the verbal, not mathematical definitions of the term "buffering" suggest that buffering strength should be positively correlated with the fraction of added H^+ ions that is neutralized by reacting with OH^- ions, rather than remaining "free". In the acidic range, say at pH 3, only a vanishing fraction reacts with OH^- ions (~1/100,000,000 or 0.000,001%), and virtually all added H^+ ions remain free. At pH 11, the chemistry is radically different: virtually all of the added H^+ ions react with OH^- (99.999999%), with a vanishing change in free H^+ concentration. The buffering ratio B directly and correctly reflects this basic process, with values of B=100,000,000 at pH 11, and B=0.000,000,010 at pH 3, respectively. Relying on the buffering value β_H+, however, one would find that the magnitudes of H^+ buffering at pH 3 and pH 11 are identical.

Koppel and Spiro's measure of buffering strength

Eight years before Michaelis and Van Slyke, Koppel and Spiro had introduced a different, first quantitative measure of buffering strength [5] which they defined as $P = dS/dpH - dS_o/dpH$. Herein, S stands for strong acid required to produce an

incremental pH change in a given sample, and S_o stands for the amount of strong acid required to produce the same pH change in pure water. By definition, the unit P will invariably yield $P = 0$ for pure water with added strong acid or base. Thus, the unit P is thus not suited to quantitate H^+ buffering strength in pure water.

The comparison with Van Slyke's and with Koppel and Spiro's approaches shows that the particular buffering strength unit hase a striking impact on the perception and quantitative description of acid-base buffering in water.

References

1. LJ Henderson: **Das Gleichgewicht zwischen Basen und Säuren im tierischen Organismus.** *Ergebnisse der Physiologie* 1909, **8:** 254-325.
2. DD Van Slyke: **On the measurement of buffer values and on the relationship of buffer value to the disociation constant of the buffer and the concentration of the buffer solution.** *J Biol Chem* 1922, **52:** 525-570.
3. PA Stewart: **Modern quantitative acid-base chemistry.** *Can J Physiol Pharmacol* 1983, **61:** 1444-1461.
4. ET Urbansky, MR Schock: **Understanding, Deriving, and Computing Buffer Capacity.** *J Chem Ed* 2000, **77:** 1640-1644.
5. A Roos, WF Boron: **The buffer value of weak acids and bases: origin of the concept, and first mathematical derivation and application to physico-chemical systems. The work of M. Koppel and K. Spiro (1914).** *Respir Physiol* 1980, **40:** 1-32.
