# Supplementary material for: The quantitation of buffering action II. Applications of the formal & general approach
Source: Theor Biol Med Model. 2005 Mar 16;2:9. doi: 10.1186/1742-4682-2-9 (PMC1079954; doi:10.1186/1742-4682-2-9)
Supplement: Additional File 3 — Other Conservative Buffered Systems [file 1742-4682-2-9-S3.pdf]

Theoretical Biology and Medical Modelling

Research

The quantitation of buffering action. II. *Applications of the formal and general approach.*

Bernhard M. Schmitt

Supplement 3:

Other Conservative Buffered Systems

Conservative buffered systems involving ligand binding.

Binding and debinding reactions stabilize the free concentration of numerous solutes. Examples include binding proteins for various hormones such as transthyretin [1], sex hormone binding globulin [2], corticosteroid binding proteins [3,4], growth hormone binding protein [5], the soluble leptin receptor [6,7], IGF-binding proteins [8], or proteins that bind exogenous, possibly toxic substances such as quinidine or vanadate [9,10]. Importantly, binding and debinding is also crucial for the transport and storage of oxygen and carbon dioxide [11,12]. The extent of buffering is sometimes considerable, and manipulating the concentration of the respective buffers or "binding proteins" will have clear physiological or pathophysiological consequences. All such processes are immediately amenable to analysis in terms of conservative buffered systems and the buffering parameters t, b, T, and B, even though binding may obey different quantitative relations as the simple Langmuir-like behavior explored in this section.

Conservative buffered systems that do *not* involve ligand binding

Our buffering concept and units are suited to quantitate H^+ buffering in pure water and in solutions of weak acids. However, they can be applied readily to phenomena that are brought about by mechanisms different from those prevailing in classical acid-base buffering. Two examples shall demonstrate that even simple chemical systems can display "perfect buffering" or even "amplification". Furthermore, we try to sketch out how the concept of conservative buffered systems may serve to quantitate "redox buffering", a phenomenon for which no satisfactory buffering strength unit seems available to date.

"Perfect buffering" - a solute beyond its solubility *(Figure 1A)*

Consider a solution of KCl in water. We may represent the total amount of KCl by the independent variable, the amount of dissolved KCl

by the transfer function, and the amount of undissolved KCl by the buffering function:

$$\begin{pmatrix} x \\ \tau(x) \\ \beta(x) \end{pmatrix} \leftrightarrow \begin{pmatrix} [KCl]_{total} \\ [KCl]_{dissolved} \\ [KCl]_{undissolved} \end{pmatrix}.$$

Formally, this system constitutes a conservative "buffered system", but one that is not related to acid-base chemistry.

The qualitative behavior of this conservative system is easy to predict *(Figure 1A)*: Before the solubility product of KCl is reached, all of the added KCl will dissolve, reflected by a transfer function $\tau(x)$ that increases with a slope of $\tau'(x)=1$. Accordingly, the buffering ratio B equals 0. Sharply beyond the solubility product, added KCl will not dissolve at all; rather, a slurry of undissolved KCl crystals will form on the bottom. Consequently, the slope of the transfer function will equal zero from here on. Accordingly, the buffering ratio B approaches infinity. Thus, this system behaves in a binary, all-or-nothing way in terms of buffering: zero buffering ($B=0$) below the solubility product or perfect buffering ($B\rightarrow\infty$) beyond that point. Interestingly, buffering strength *increases* from weak to strong with increasing solute concentration. In contrast, the finite-capacity buffering by Langmuir buffers (see *Figure 1* of the main text of *Buffering II*) *decreased* from strong to zero buffering with increasing solute concentration.

This binary type of buffering behavior *(Figure 1A)* is observed with any solute that exhibits a solubility maximum. The underlying principle is exploited, for instance, in the design of reference electrodes for pH meters: Here, evaporation of water tends to increase the concentration of KCl in the reference solution. By setting initial KCl concentration to the solubility maximum, however, KCl concentration becomes perfectly stabilized, and the undesired consequences of evaporation are prevented. The concentration of mercury in calomel electrodes is stabilized analogously. Herein, the concentration at which perfect buffering of mercury occurs can be adjusted -within certain limits- by changing the concentration of KCl. In all these cases, it seems easily justifiable to refer to the stabilization of the respective ion concentrations as "buffering" in the full meaning of that word, and to use our buffering parameters t, b, T, and B in order to quantify the particular degree of that stabilization.

Thermal buffering – cold drink in a cooler box *(Figure 1B)*

The concept of a "buffered system" and the buffering parameters t, b, T, and B are applicable not only to phenomena involving ion concentrations, but other quantities as well. In this example, the quantity of interest has the dimension of a heat energy Q, and the physical system consists of an insulated box containing a cold pack (i.e., a generic cooler) plus a cup containing a cold drink that is in thermal equilibrium with the ice pack *(Figure 1B)*.

We represent this system mathematically as a "buffered system" by assigning the roles of independent variable, transfer function and buffering function to total heat energy inside the cooler, and the individual heat energies in cold pack and cold drink, respectively (all other heat energies inside the box are considered negligibly small):

$$\begin{pmatrix} x \\ \tau(x) \\ \beta(x) \end{pmatrix} = \begin{pmatrix} Q_{Total} \\ Q_{Drink} \\ Q_{ColdPack} \end{pmatrix}$$

Addition of heat energy to the system by convection (open lid) or by heat conduction (less than perfect insulation) increases total heat energy by a certain amount ΔQ_{Total}. The additional energy is absorbed in part by the drink, in part by the cold pack. The particular proportion between these two fractional heat flows is determined by the individual heat capacities, i.e., the product of mass and specific heat capacity. Without cold pack, ΔQ_{Total} is entirely deposited in the drink and warms it up. The buffering coefficient without cold pack is

$$b = \frac{dQ_{Coldpack}}{dQ_{Total}} = 0 \quad ,$$

and the buffering ratio is

$$B = \frac{dQ_{Coldpack}}{dQ_{Drink}} = 0 \quad .$$

We can say, the drink is not buffered at all against inflowing heat, and this is appropriately reflected in the two measures of buffering strength. With a cold pack, for instance one of the same mass and specific heat capacity as the drink, half of the added energy ΔQ_{Total} would flow into the cold pack, and the drink would warm up half as much as without cold pack. We can say that the drink is buffered against inflowing heat. The extent of this buffering can be specified unambiguously by the parameters b or B. In this case, they assume finite, positive values of b=0.5 and B=1. The number of cold packs is reflected faithfully and intuitively by the buffering ratio B: Doubling the number of cold packs will double B, fivefold number of cold packs results in a five times higher value of B, etc.. Physically, heat energy is conserved in this example. Mathematically, this gives rise to a conservative two-partitioned system, the simplest form of partitioned systems *(Buffering I)*.

Other conservative buffered systems not involving ligand-binding

Numerous other systems that involve mechanisms other than ligand binding can be analyzed under the aspect of "How much of a conserved quantity goes to its destination, and how much goes elsewhere". Such systems can be represented by conservative two-partitioned systems, analogous to the "free vs. bound" electrolytes in a given compartment. Their buffering behavior can then be described quantitatively with the aid of the parameters t, b, T, and B. Examples include phenomena of physiological interest (e.g. the distribution of drugs and toxic substances between "target" and "non-target" body compartments, or the distribution of blood flow between various organs as elaborated below), physical processes (e.g. the efficiency of energy transduction by engines, "humidity buffering" in buildings, "transmittance" vs. "reflectance" and "absorbance" of light), economical (e.g. the actual allocation of an allotted economic aid to "earmarked" vs. "unintended" purposes; the apportionment of gross income into "net income" vs. "taxes and other dues" which are non-linear functions of gross income under a tax regime with progressive rates), and so on. The formal framework established in the preceding article *(Buffering I)* together with the examples in the present one provide the tools required to formalize such "non-classical" manifestations of conservative buffered systems appropriately.

„Amplification" in an electrolyte solution – strong mineral acid *(Figure 1C)*

A simple "buffered system" displaying yet another type of buffering behavior is constituted by a solution of a strong mineral acid in water, e.g. hydrochloric acid. We formalize this system in the following way: The independent variable x represents the total concentration of added strong acid, $[HCl]_{total}$; the transfer function $\tau(x)$ stands for H^+ ion activity $\{H^+\}$; and the buffering function $\beta(x)$ denotes the difference $[HCl]_{total}$ - $\{H^+\}$:

$$\begin{pmatrix} x \\ \tau(x) \\ \beta(x) \end{pmatrix} \leftrightarrow \begin{pmatrix} [HCl]_{total} \\ \{H^+\} \\ [HCl]_{total} - \{H^+\} \end{pmatrix}.$$

Herein, the buffering function $\beta(x)$ lumps together the effect of various chemical processes that deflect the ion activity coefficient away from unity. With $\tau(x)+\beta(x)=x$, the system is conservative by design.

As a thermodynamic quantity, "ion activity" can be measured exactly in a number of ways, although it cannot be derived rigorously from first physico-chemical principles to date, in spite of theoretically founded approximations [13]. Thus, the following analysis is a good example that buffering is a quantitative pattern, as opposed to a particular chemical mechanism. As such, buffering can be fully quantitated empirically, without the aid of any knowledge about its "nature" or mechanistical underpinnings that would allow for analytical solutions.

The ratio $\gamma_{H^+} = \{H^+\}/[H^+]$, termed "mean hydrogen ion activity coefficient", is known to decrease, probably due to ion pair formation, from $\gamma_{H^+} = 1$ at $[HCl] = 0$ mM to $\gamma_{H^+} \approx 0.8$ at $[HCl] = 1$ M. As $[HCl]$ increases further, γ_{H^+} increases again progressively, probably due to capture of water in ion hydration shells and electrostatic repulsion

Figure 1: Some conservative buffered systems.

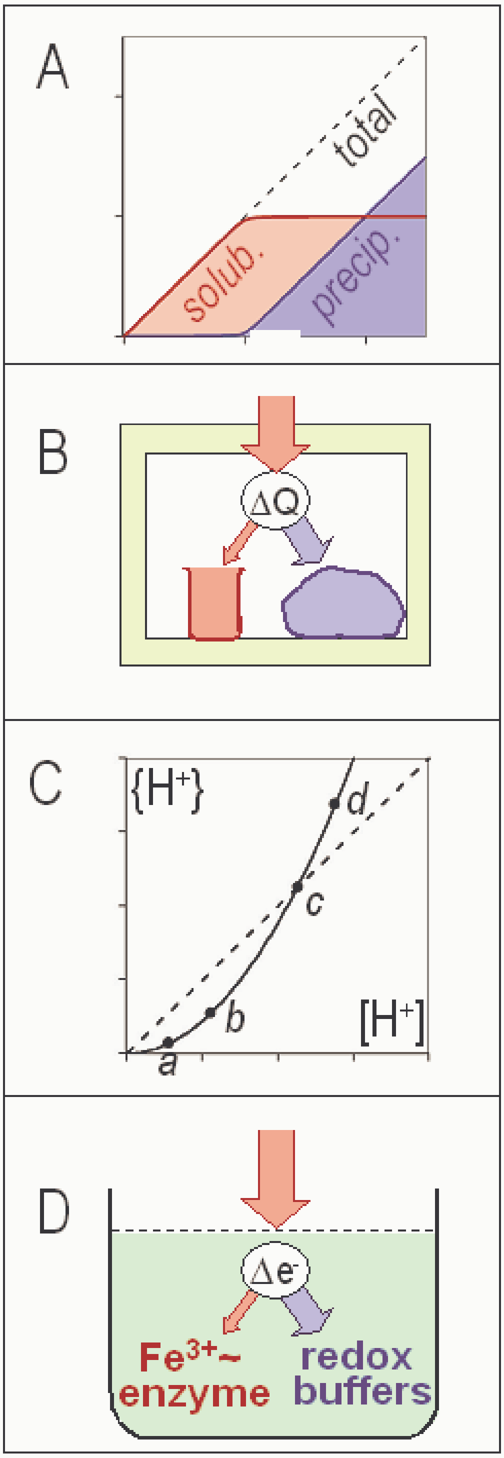

A, Buffering of free solute concentration by precipitation.

Schematic plot of total solute *(total)*, solubilized solute *(sol.)*, and precipitated solute *(precip.)* as functions of total solute *(abscissa)*. Equal units on both axes. When the solubility maximum is reached, the system changes from zero buffering to virtually infinite buffering.

B, Buffering of heat energy.

Heat energy added (ΔQ) to a thermally isolated system is absorbed in part by a cold drink *(left, red)* and by cold pack *(right, blue)* which have the same temperature; the cold pack acts as a thermal „buffer". Thermal buffering strength can be determined from the proportion between the respective fractions of ΔQ that are absorbed by the two masses.

C, Buffering of H^+ ion activity in a solution of a strong mineral acid.

Schematic plot of H^+ ion activity $\{H^+\}$ against added H^+ ions $\Delta[H^+]$. Units on both axes approximately equal to 1 mole/liter. *a,* moderation: one added H^+ ion increases $\{H^+\}$ by less than one. Transfer coefficient $t<1$; *b,* zero buffering: one added H^+ ion increases $\{H^+\}$ by exactly one, and $t=1$; *c and d*, amplification: one added H^+ ion increases $\{H^+\}$ by more than one, and $t>1$; *c,* the unique point where ion activity and ion concentration are identical: $\{H^+\} = [H^+]$.

D, Buffering of an enzyme's oxido-reduction state by redox buffers.

Free electrons (Δe^-) added into a solution will bind in part to the oxidized form of an iron enzyme (Fe^{3+}~enzyme) and in part to other compounds present in the solution (redox buffers). Redox buffering strength can be determined from the proportion between the respective fractions of Δe^- that are absorbed by the two classes of compounds.

between ions. Around $[HCl] = 2\,M$, the coefficient γ_{H+} thus reaches again 1 and subsequently surpasses this value significantly. For instance, γ_{H+} is ~2 at $[HCl] = 5\,M$, ~4 at $[HCl] = 5\,M$ and ~200 at $[HCl] = 12\,M$ [14]. In a solution of a strong acid in water, hydrogen ion *activity* is thus stabilized to a variable extent against the addition or removal of strong acid, depending on total H^+ concentration *(Figure 2C)*. Again, such a situation fully justifies to speak of buffering and to employ our buffering parameters in order to quantitate the precise degree of that stabilization.

When we write H^+ ion activity as a function $\gamma_{H^+}([HCl])$ of strong mineral acid concentration $[HCl]$, the transfer coefficient t as a function of $[HCl]$ is given as:

$$\begin{aligned} t([HCl]) &= \frac{d}{d[HCl]} \times \{H^+\} \\ &= \frac{d}{d[HCl]} \times \gamma_{H^+}([H^+]) \cdot [H^+] \\ &= \frac{d}{d[HCl]} \times \gamma_{H^+}([H^+]) + \gamma_{H^+}([H^+]) \end{aligned} .$$

Thus, the transfer coefficient is different from the ion activity coefficient γ_{H+}. Likewise, we may compute the other buffering parameters for such a solution. We will then find "non-inverting moderation" (i.e., $0 \le t \le 1$) up to $[HCl] \approx 1\,M$. Beyond that concentration, γ_{H+} and $t(x)$ will increasingly exceed 1, implying that buffering coefficient b and buffering ratio B assumes negative values. "Negative buffering", or, more systematically, "non-inverting amplification", may appear an unfamiliar concept, but follows strictly from our general definition of these parameters *(Buffering I)*. The terms "negative buffering" and "amplification" also make sense intuitively: every HCl molecule added increases the number of "thermodynamically active" H^+ ion by *more than one*. Considering the entire concentration range, strong electrolyte solutions are "combined buffers/amplifiers".

Evidently, the parameters t, b, T, and B do not "explain" anything. They do no more -and no less- than describing quantitatively the buffering behavior of this system.

Redox buffering

Similarities between redox buffering and H^+ buffering

Just as protons do, electrons bounce from molecule to molecule on their trajectory towards the lowest energy level available. Moreover, similar to protons, the degree of association of electrons with certain biomolecules affects a multitude of cellular functions. Herein, the individual redox states of individual chemical species are in principle completely determined by a single variable, the oxido-reduction potential E_h. This dependence on E_h is a homolog of the "isohydric principle" *(Buffering I – Supplement 9)* which states that the protonation-deprotonation equilibrium at any particular proton binding site in a given physico-chemical compartment is completely constrained by the concentration of free protons, but does not depend on other H^+ binding sites present. The importance of oxidation-reduction processes is widely recognized by now, although this view gained acceptance rather belatedly as compared to the early appreciation of the relevance of acid-base balance. Just as organisms need to keep the degree of *protonization* of various biomolecules within certain limits, organisms also need to control the degree of *"electronization"* of other biomolecules. For protons and electrons alike, this control involves active mechanisms that adjust the system to a desired setpoint, as well as passive "buffering" mechanisms that minimize the deviations from the setpoint in response to disturbances.

The dichotomy „bound" vs. „free" does not work for electrons

The technical challenges of determining experimentally the redox potentials and the concentrations of the contributing molecular species exceed those encountered in acid-base chemistry. For instance, the assumption of complete equilibrium is harder to assure, and the solvent plays a different role than in acid-base buffering: Even though the equilibria of many redox reactions depend on $[H^+]$ (or pH), water itself does not

provide a good experimental "read-out" of the system's redox state.

In the accompanying article *(Buffering I)*, we found that the use of buffering terminology and units presupposes a certain dichotomous structure. Namely, it is required to lump the various observed quantities into two separate groups, a quantity that is being buffered (e.g. free H^+ ions), and another one that reflects buffering (e.g. bound H^+ ions). A similar dichotomy is also nedessary to get a grip on redox buffering. The distinction between "free" and "bound" electrons, however, does not work here inasmuch there are no "free" electrons (similarly, there are no "free" *protons* in the strict sense; the important point is, however, that the distinction between water-bound and buffer-bound protons is conceptually and technically straightforward). Neither does the equilibrium between the system under study and an *external* reference electrode (i.e., the standard oxido-reduction potential E_h) induce such a dichotomy within the system itself. In our view, one cannot meaningfully speak about buffering in such a system.

The slope of the redox titration curve tells nothing about the partitioning of electrons

Accordingly, we think that there are problems associated with a recently suggested "redox buffer strength" unit [15,16] that was based on E_h by analogy with Van Slyke's pH-based buffering strength unit. When titrating a solution's E_h with a strong reductant R and plotting E_h against [R], the value of de Levie's dimensionless unit is proportional to the slope of the titration curve. On the one hand, this unit provides an unequivocal quantitative measure for the behavior of a redox system. Our criticism is that what we might want to know when we ask about "redox buffering" is not the electrical potential of the system as a whole with respect to a standard reference, but rather how electrons distribute internally between different electron acceptors, analogous to the partitioning of protons between water and buffer molecules. To illustrate that point, assume that we are interested in a particular redox-sensitive enzyme that becomes inactive upon reduction.

Firstly, consider two solutions with identical "redox buffer strength B" according to de Levie, one containing nothing but a fixed concentration $[E]_{total} = c$ of the redox-sensitive enzyme, and another one containing half the amount of enzyme, i.e., $[E]_{total} = c/2$, plus an equal amount of a second redox compound, $[F]_{total} = c/2$, with the same electrochemical properties as the enzyme E. Then, the responses of the entire systems' reduction potential E_h to addition of strong oxidant or reductant will be identical. The number of electrons binding to the enzyme of interest, however, and the absolute decrease of enzyme activity will differ.

Conversely and secondly, it is easy to design enzyme solutions that have different redox buffer strength B according to de Levie's unit, but do not differ with respect to the changes of enzyme activity in response to titration with a reductant. In one extreme case, the reduction potential E_h may be stabilized perfectly by a high concentration of enzyme, but still every single reducing equivalent added finds its way to a copy of the enzyme and inhibits it – in other words, the enzyme is not buffered at all against redox titrants.

The example shows that the "stabilization of the concentration of reduced enzyme" may deviate in any unforeseeable manner from the "stabilization of the redox potential". Usually, the parameter of interest is the redox state of a particular enzyme or other biologically active molecule. Adopting such a perspective, a redox buffering strength unit that is based on the redox potential in itself appears as a surrogate parameter of questionable validity.

The buffering measures (t, b, T, B) as measures of redox buffering strength *(Figure 1D)*

Viewing redox reactions in terms of a two-partitioned system

In this sense, a more valid measure of redox buffering strength can be obtained using our definition of buffered systems and the parameters t, b, T, and B to redox systems. As with all buffered systems, the crucial point herein is to assign the complementary roles of transfer function and buffering function in a meaningful way to two appropriate chemical parameters.

For instance, we may be interested in the redox state of iron that is bound to the heme group of a particular enzyme ("EFe"), with EFe^{2+} and EFe^{3+} as the major species involved in the equilibrium. To probe how well the redox state of that enzyme is "buffered" within a given system, we titrate the system up or down by adding or removing electrons (e^-) in the form of strong reducing or oxidizing agents, respectively. A certain amount of strong titrant changes the total concentration of electrons within the original system by an equal amount $\Delta[e^-]_{Total}$; furthermore, we denote the concentration of electrons bound to the enzyme's heme-iron by $[e^-]_{Enzyme}$, and the concentration of electrons bound elsewhere by $[e^-]_{Others}$. Then, we can formulate a conservative buffered system:

$$\begin{pmatrix} x \\ \tau(x) \\ \beta(x) \end{pmatrix} \leftrightarrow \begin{pmatrix} \Delta[e^-]_{Total} \\ \Delta[e^-]_{Enzyme} \\ \Delta[e^-]_{Others} \end{pmatrix}.$$

Computing the four buffering parameters (t, b, T, B) for a redox system

Such a system is illustrated in *Figure 1D*. The four buffering parameters in this system follow as usual:

$$t = \left(\frac{d[e^-]_{Enzyme}}{d[e^-]_{Total}} \right) \leftrightarrow \left(\frac{d[EFe^{2+}]}{d[reductant]} \right) \leftrightarrow \left(\frac{d[EFe^{3+}]}{d[oxidant]} \right)$$

$$b = \left(\frac{d[e^-]_{Others}}{d[e^-]_{Total}} \right) \leftrightarrow \left(1 - \frac{d[EFe^{2+}]}{d[reductant]} \right) \leftrightarrow \left(1 - \frac{d[EFe^{3+}]}{d[oxidant]} \right)$$

$$T = \left(\frac{d[e^-]_{Enzyme}}{d[e^-]_{Others}} \right) \leftrightarrow \left(\frac{d[EFe^{2+}]}{d[reductant] - d[EFe^{2+}]} \right) \leftrightarrow \left(\frac{d[EFe^{3+}]}{d[oxidant] - d[EFe^{3+}]} \right)$$

$$B = \left(\frac{d[e^-]_{Others}}{d[e^-]_{Enzyme}} \right) \leftrightarrow \left(\frac{d[reductant] - d[EFe^{2+}]}{d[EFe^{2+}]} \right) \leftrightarrow \left(\frac{d[oxidant] - d[EFe^{3+}]}{d[EFe^{3+}]} \right)$$

These equations define the buffering parameters **t**, **b**, **T**, and **B** rigorously for the particular selection of buffered variable vs. buffering variable, i.e., $[e^+]_{Enzyme}$ vs. $[e^+]_{Others}$. Note that we changed the question from the original "How well is redox potential E_h stabilized" to "How well is the concentration of EFe^{2+} stabilized".

Practical aspects

Biologically relevant redox systems are usually complex mixtures of a variety of potential electron donors and acceptors and thus constitute what we have termed "multi-partitioned systems" *(Buffering I)*. Thus, many different two-partitioned and buffered systems can be derived from one such system that are formally correct and potentially meaningful. For instance, when one studies the stability or instability of the cytoplasmic Fe^{2+} level, one should use cytoplasmic Fe^{2+} as the transfer function and then treat the heme-iron from our previous example as part of the buffer compartment. Clearly, "$[EFe^{2+}]$ buffering" may differ in magnitude in any possible way from "cytoplasmic $[Fe^{2+}]$ buffering, and either definition of redox buffering strength may be useful and meaningful.

Experimentally, the two indices of this redox buffering strength, buffering coefficient b and buffering ratio B, can be determined in a straightforward way by redox titration. Herein, at least two out of the three variables (total, reduced, and oxidized redox compound) are measured individually. In fact, this approach may be less challenging technically than to determine E_h.

Autoregulation of flow in the face of variable total flow

Circulation with one organ – zero buffering

First, we derive a quantitative measure for "autoregulation" from the description of volume flow in one organ as a function of total blood flow (i.e., of cardiac output). As a first approximation, we model the parallel arrangement of organs in the circulation by a parallel arrangement of rigid cylindrical tubes (see *Figure 2* of the main text of *Buffering II*). In the simplest case, the number of parallel tubes is one, corresponding to a single organ

1 in the circulation (e.g. the brain). Total flow then equals the flow in the single tube, or $\Phi_{total}=\varphi_1$.

We may omit various specific details of this system in order to emphasize its quantitative aspects. Thus, we reduce the circulatory system to a "black box" with a single input Φ_{total} and a single output φ_1. In signal processing terminology, the black box constitutes a "transfer element" or "transducer", and the relationship between input x and output y a "transfer function" $y=\tau(x)$. In this example, the transfer function is of the type $y=a\times x$ with $a=1$.

Circulation with two similar organs in parallel – linear buffering

Next, consider a circulation comprising two similarly sized, parallel tubes 1 and 2. They can be interpreted as "organs", e.g. as brain and muscles, respectively. The variables are total flow Φ_{total} and two partial flows φ_1 and φ_2. The coefficient a in the equation $\varphi_1=a\times x$ has now dropped to $a=0.5$. As volume flow is a conserved quantity, the two outputs are not independent. Rather, "muscle perfusion" is determined by total flow and cerebral blood flow as $\varphi_2=\Phi_{total}-\varphi_1$. We represent φ_2 explicitly as a second output z of the transfer element. Importantly, the resulting "single-input, dual-output system" can be represented by an ordered pair of functions $\{y=\tau(x),\ z=\beta(x)\}$, i.e., by a "buffered system". The two functions can be ordered in two ways; if we are interested in how much cerebral blood flow is buffered against total flow, we would form the following system:

$$\begin{pmatrix}\Phi_{total}\\ \varphi_1\\ \varphi_2\end{pmatrix}\leftrightarrow\begin{pmatrix}\Phi_{total}\\ \varphi_{brain}\\ \varphi_{muscle}\end{pmatrix}\leftrightarrow\begin{pmatrix}\text{input x}\\ \text{output y}\\ \text{output z}\end{pmatrix}\leftrightarrow\begin{pmatrix}x\\ \tau(x)\\ \beta(x)\end{pmatrix}.$$

These systems are formally equivalent, which implies that the four buffering parameters (t, b, T, and B) are defined unambiguously and can be interpreted in a meaningful way on various levels of abstraction, including the levels of *i)* combinations of pure mathematical functions, *ii)* generic transfer elements, *iii)* hydraulic networks, or *iv)* cardiovascular circulations. On the latter level, buffering coefficent b and buffering ratio B can serve as valid measures of autoregulation (here: "autoregulation of brain perfusion"). Herein, the buffering ratio B again yields a genuine ratio scale. For the buffered system corresponding to the two parallel tubes in our second example, we find that buffering is linear (b=const.=0.5), dimensionally homogeneous ([x]=[y],[z]), and conservative [$\sigma'(x)=\tau'(x)+\beta'(x)=1$]. Expressing input vs. outputs in different units of the same physical dimensions (e.g., x in ml/sec vs. y,z in ml/min) results in an equivalent buffered system that is again dimensionally homogeneous ($[x]=[y],[z]=L^3/T$) and conservative.

Circulation with two organs in parallel – non-linear buffering

To make the model more realistic, we allow cerebral and muscular blood flows to vary non-linearly with total blood flow. In the model, non-linearity is achieved by replacing at least one of the rigid tubes by an elastic one. Hydraulic conductivity L_P is no longer constant now: Under conditions of higher total flow (i.e., at higher pressure), the hydraulic conductivity of an elastic tube will increase in some non-linear way, and so will the fraction of total blood flow in this branch of the circulation. The sum of both partial flows again equals total blood flow, i.e., the system is conservative and dimensionally homogeneous. On the other hand, the presence of elastic tubes in the physical model turns the corresponding buffered system into a non-linear buffered system (b≠const.). Using elastic tubes that exhibit the appropriate pressure-dependence of hydraulic conductivity, all possible manifestations of autoregulation in this sense can thus be modelled physically.

Interestingly, when autoregulation is defined via the relationship between total flow and partial flow in a particular branch, autoregulation may be observed even when the "autoregulated" compartment is in fact is a rigid tube (see lower left of *Figure 2C* of the main text of *Buffering II*) in which any modification of the total flow-partial flow relationship completely depends on parallel shunts. Therefore, autoregulation in this sense cannot be studied in the isolated organ.

References

1. G Schreiber: **Beyond carrier proteins: The evolutionary and integrative roles of transthyretin in thyroid hormone homeostasis.** *J Endocrinol* 9999.
2. W Rosner: **Errors in the measurement of plasma free testosterone.** *J Clin Endocrinol Metabol* 1997, **82:** 2014-2015.
3. SL Alexander, CHG Irvine: **The effect of social stress on adrenal axis activity in horses: the importance of monitoring corticosteroid-binding globulin capacity.** *J Endocrinol* 1998, **157:** 425-432.
4. DH Jennings, MC Moore, R Knapp, L Matthews, M Orchinik: **Plasma steroid-binding globulin mediation of differences in stress reactivity in alternative male phenotypes in tree lizards, Urosaurus ornatus.** *Gen Comp Endocrinol* 2000, **120:** 289-299.
5. JD Veldhuis, ML Johnsons, LM Faunt, M Mercado, G Baumann: **Influence of the high-affinity growth hormone (GH)-binding protein on plasma profiles of free and bound GH and on the apparent half-life of GH. Modeling analysis and clinical applications.** *J Clin Invest* 1991, **91:** 629-641.
6. FMH van Dielen, C van't Veer, WA Buurman, JWM Greve: **Leptin and soluble leptin receptor levels in obese and weight-losing individuals.** *J Clin Endocrinol Metabol* 2002, **87:** 1708-1716.
7. V Ogier, O Ziegler, L Mejean, JP Nicolas, A Stricker-Krongrad: **Obesity is associated with decreasing levels of the circulating soluble leptin receptor in humans.** *Int J Obes Relat Metab Disord* 2002, **26:** 496-503.
8. BLG Nyomba, L Berard, LJ Murphy: **Free insulin-like growth factor I (IGF-I) in healthy subjects: relationship with IGF-binding proteins and insulin sensitivity.** *J Clin Endocrinol Metabol* 1997, **82:** 2177-2181.
9. JH Li, JQ Xu, XM Cao, L Ni, Y Li, YY Zhuang, JB Gong: **Influence of the ORM1 phenotypes on serum unbound concentration and protein binding of quinidine.** *Clin Chim Acta* 2002, **317:** 85-92.
10. G Heinemann, B Fichtl, M Mentler, W Vogt: **Binding of vanadate to human albumin in infusion solutions, to proteins in human fresh frozen plasma, and to transferrin.** *J Inorgan Biochem* 2002, **90:** 38-42.
11. M Nikinmaa: **Oxygen and carbon dioxide transport in vertebrate erythrocytes: an evolutionary change in the role of membrane transport.** *J Exp Biol* 1997, **200 (Pt 2):** 369-380.
12. IW McIntyre, KL Campbell, RA MacArthur: **Body oxygen stores, aerobic dive limits and diving behaviour of the star-nosed mole (Condylura cristata) and comparisons with non-aquatic talpids.** *J Exp Biol* 2002, **205:** 45-54.
13. P Debye, E Hückel: **Zur Theorie der Elektrolyten.** *Phys Z* 1923, **24:** 185.
14. SK Lower. Acid-base equilibria and calculations. 1997. http://www.chem1.com/acad/pdf/c1xacid2.pdf.
15. R de Levie: **Redox Buffer Strength.** *JChemEd* 1999, **76:** 574-577.
16. R de Levie: **A simple expression for the redox titration curve.** *J Electroanal Chem* 1992, **323:** 347-355.
