# Supplementary material for: The quantitation of buffering action II. Applications of the formal & general approach
Source: Theor Biol Med Model. 2005 Mar 16;2:9. doi: 10.1186/1742-4682-2-9 (PMC1079954; doi:10.1186/1742-4682-2-9)
Supplement: Additional File 5 — Notes on Time-Dependent Buffering or "Muffling" [file 1742-4682-2-9-S5.pdf]

Theoretical Biology and Medical Modelling

Research

The quantitation of buffering action. II. *Applications of the formal and general approach.*

Bernhard M. Schmitt

Supplement 5:

Notes on Time-Dependent Buffering or "Muffling"

The effect of test stimulus duration.

Our definition of muffling strength rests on the assumption that the initial deviation e_0 is instantaneous On the theoretical level, there are indeed no problems with this definition, which is simple, rigorous, and free of any arbitrary bias. In practice, however, a finite time T is usually required to bring about a deviation e_0. Therefore, it is necessary to consider the potential problems caused by this deviation from ideality. Such a finite "rise time" introduces an additional variable. Rise time may be relatively short, e.g. with caged compounds that are released by a flash of light (either ligands or buffers), or relatively long, e.g. with iontophoretic injection of ions. The addition of e_0 free molecules will increase the total number $n(t)$ of free molecules by an identical amount e_0 only if it occurs instantaneously. In contrast, when the same number e_0 of molecules is released over a finite time T, then muffling action will reduce the peak amplitude Δn to a value $\Delta n < e_0$ (assuming nonnegative muffling power).

As a general rule, the apparent muffling ratio $M_{app}(t)$ determined experimentally for a time t and with a stimulus of finite duration T will only provide good estimates of the true muffling ratio $M(t)$ if stimulus duration T is negligible compared to integration time ($T << t$). Otherwise, positive muffling will overestimated. Consequently, to determine muffling $M(t)$ for a given time integration time t, stimuli must be chosen that bring about the complete perturbation within a negligibly short fraction of t. Reversly, a given stimulus only serves to quantitate muffling on time scales that considerably exceed its own duration. Obviously, muffling cannot be judged before the stimulus has been applied completely ($t < T$). For a given time scale $t > T$, the overestimation of the muffling ratio $M(t)$ is particularly serious for intermediate values of $M(t)$ where the rate of delivery (e_0/T) on the one hand, and the rates of buffering or transport on the other, are similar. The experimental error will be small if the true muffling ratio $M(t)$ is either very large (because the system practically reaches equilibrium) or very small (because both true and estimated muffling ratio approches zero).

Problems with previous units of "muffling strength"

Transient amplitude alone cannot define muffling strength.

Interestingly, the few previous attempts to quantitate muffling action have relied exclusively on the peak amplitude of the transient that is elicited by an acute ion load, either implicitly [1] or explicitly in the definitions of a „Ca^{++} muffling ratio" and „Ca^{++} muffling power" [2]. In our view, these units suffer from two serious flaws that make them unreliable and potentially misleading.

Firstly, "peak size" alone is not reproducibly correlated with muffling unless stimulus duration is standardized. Secondly, even if stimulus duration could be defined and standardized unambiguously, peak size is "blind" to all other parameters of the ion concentration transients which clearly reflect important aspects of binding and transport combined, i.e., of "muffling" proper. To illustrate that point, consider two extremes of a calcium injection time course.

Infinitely slow injection. With infinitely slow injection, there will be no peak at all. We can calculate the corresponding muffling power and muffling ratio according to Schwiening & Thomas [2], with the variables denoted according to the conventions introduced in this article. Their muffling ratio M_r is given as

$$\text{muffling ratio } M_r = \frac{\Delta x}{\Delta e_{max}} ,$$

where Δx denotes the number of added free Ca^{++} ions, and Δe_{max} the maximum of the deviation $e(t)$ of the number of free calcium ions from their baseline value, as observed during the peak. Here, there is no peak, and thus $\Delta e_{max}=0$, resulting in a muffling ratio that is infinitely large. Their muffling power, on the other hand, is given as

$$\text{muffling power} = \frac{\Delta x}{-\Delta \log(n)} ,$$

where x and n must be expressed in multiples of 1 mole and be normalized with respect to a reference volume of 1 liter. Then, $-\Delta\log(n)$ analogously denotes the maximum deviation of the pCa from baseline as observed during the peak. In the absence of a peak, the denominator is again 0, and muffling power becomes infinitely large.

Infinitely fast injection. If the same number e_0 of free calcium ions is released instantaneously, the total number of free calcium ions will increase instantaneously by

$$\Delta e = e_0 .$$

Under these conditions, we find that

$$\Delta x = e_0 = \Delta e_{max} ,$$

and

$$-\Delta\log(n) = \log\frac{n_0}{n_0 + e_0} .$$

Consequently, the „calcium muffling ratio" will always equal 1, and the „calcium muffling power" will equal $\dfrac{e_0}{\log\dfrac{n_0}{n_0+e_0}}$.

Notably, these muffling indices could be computed correctly for these two limiting cases without any knowledge or assumptions about binding or transport in the system under study. In other words, these indices of muffling power become completely dissociated from the actual speed and completeness with which the initial disturbance is subsequently counteracted within the system. Ironically, this problem arises even when the experimental modalities are "too good", i.e., when the injection of calcium is very efficient.

Paradoxically, muffling ratio and muffling power may thus become insensitive to the very essence of what researchers originally intended to capture when they coined the term „muffling", namely the combined action of local chemical buffering, diffusion of ions and ion-buffer complexes, and of membrane transport [1]. Likewise, these two muffling units fail to discriminate between the four grossly different scenarios illustrated in *Figure 3B* of the main text of *Buffering II*.

The underlying problem with these two muffling units is their claim to constrain muffling strength completely by a single time-independent parameter. As shown above, a particular peak size can be associated with very different overall trajectories of

Ca^{++} concentration, and thus with very different overall "exposure" of the cytoplasm to free Ca^{++}.

To make things worse, even the direction in which the peak size will change with stronger or weaker muffling is not predictable: the peak size of a concentration transient is decreased synergistically by binding and flux, whereas the speed with which $n(t)$ travels towards the new equilibrium is affected antagonistically by binding and flux [3], and better binding may actually *increase* the time-weighted "exposure" of the cytoplasm to elevated Ca^{++} concentrations.

For instance, if efflux is brought about by simple diffusion, additional binding will decrease the spatial concentration gradient ∇c, and hence also proportionally decrease the net flux $\phi = -D\nabla c$, where D is the diffusion coefficient. The effect of a buffer in such a case is largely analogous to the well-known „wind-pipe" effect of the aorta, or to the effect of a electrical capacitor.

In the first case, when volume is injected into the arterial system during the systole, the high compliance of the aorta results in blood pressure transient of smaller amplitude, but longer duration.

In the second case, a higher capacitance in parallel with a resistor will decrease the voltage change produced by the deposition of a charge, but increase its duration by slowing down the uncharging of the capacitor.

For instance, such effects were impressively apparent for calcium muffling in various neuronal cell types (*Table 1* in [5]).

The muffling ratio provides a theory-free, mechanistically unbiased measure of dynamic buffering.

The buffering and transport processes that determine steady-state levels and transients of free Ca^{++} ion concentration have been analyzed empirically and theoretically in great detail [4,5]. Steady-state buffering of Ca^{++} is now often quantitated using the "Ca^{++} binding ratio κ_S, a Ca^{++}-specific equivalent of our "buffering ratio B" (discussed in *Buffering I – Supplement 10*).

Time-dependent Ca^{++} buffering is addressed under several aspects. For instance, some approaches assume homogeneous cytoplasmic Ca^{++} concentration and model Ca^{++} influx based on instantaneous buffering and finite-rate extrusion [5]. Others focus on local Ca^{++} dynamics on a subcellular level, e.g. around the cytoplasmic opening of a Ca^{++} channel or at a synapse; the analysis of these processes needs to take into consideration parameters such as Ca^{++} buffer concentration, equilibrium constant and rates, diffusion rates of free and Ca^{++}-bound buffer species etc. and turns into a demanding task technically as well as theoretically [4].

In such studies of time-dependent buffering processes, our "muffling ratio" $M(t)$ may be employed as a further useful, purely descriptive measure. According to the specific requirements, the muffling ratio may be computed either for the entire cytoplasm of a cell, or for a small volume element of interest. Given the baseline Ca^{++} concentration and its time course upon a fast Ca^{++} ion load, the muffling ratio provides a direct measure of the efficiency with which this disturbance is counteracted in the particular volume and time window of interest.

The muffling ratio provides a useful measure even for those cases where the disturbance is not counteracted at all, or even amplified. Under such conditions, there may be no "peak", yet this does not prevent us from finding an exact numerical expression for the said efficiency.

Importantly, the validity of that measure of muffling is independent from the underlying mechanisms. In particular, the muffling ratio $M(t)$ can be used whether Ca^{++} is in equilibrium with its buffers or not, with mobile or fixed buffers, high- or low- affinity buffers, buffers with hyperbolic or with sigmoideal binding characteristics, and at any degree of buffer saturation. The validity of the parameter $M(t)$ obviously depends, however, on the accuracy with which the time course and the corresponding integrals are determined. For instance, slow equilibration of Ca^{++} with the indicator dye would result in incorrect estimates of $M(t)$, at least for small values of t.

Thus, the muffling ratio does not require any information about the underlying mechanisms. By the same token, they do not deliver any information about these parameters, and can not be applied to that end. Furthermore, the muffling ratio is not suited to measure either the information that may be encoded in the sometimes complex ion concentration changes over time („calcium signalling", "proton signalling", etc.), or the "filtering" of this information. For this reason, a direct correlation between muffling strength and particular effects downstream to the Ca^{++} signal may not exist. Rather, this general and formal measure could serve to complement the mechanistic analyses of time-dependent buffering with a condensed measure for the overall efficiency of this process.

The muffling ratio $M(t)$ is not restricted to the study of Ca^{++} dynamics, and may be applied fruitfully to numerous other processes. These include buffering and extrusion of cytoplasmic H^+ ion loads, the redistribution and excretion of ions (e.g. of K^+ ions in the interstitial spaces in the brain) or drugs injected into the extracellular space, or the binding to receptors and "decoy receptors", wash-out and re-uptake of neurotransmitters upon their release into the synaptic cleft, to name but a few.

Time-dependent buffering involving discrete variables, and periodic or stochastic events

The muffling ratio is based on integrals, and can therefore be applied directly to time courses of variables that exhibit discrete rather than continuous and differentiatable values, and to periodic or stochastic events. This applicability follows from their original definition, without any additional modifications, and without sacrificing the formal rigor inherent to that unit.

Such an approach may be needed, for instance, to capture the discrete and stochastic behavior exhibited by the number of free H^+ ions in a small, alkaline volume element (e.g., in the mitochondrial matrix) on a sufficiently fast time scale. "Steady-state concentrations" here exist only as statistical averages, whereas the actual number of free H^+ ions at any specific time -necessarily a natural number-randomly fluctuates around that average.

Likewise, the path to a new steady-state average upon an acid or base load comprises exclusively natural numbers. Thus, the functions $e(t)$ and $m(t)$ that describe error and error reduction, respectively, are discontinuous and not differentiable, as well as the corresponding integrals $\varepsilon(t)$ and $\mu(t)$. Computation of the corresponding muffling ratio is possible because it comprises only division, not differentiation. Muffling ratios from individual experiments that involve stochastic events primarily provide an empirical description of that specific experiment; generalizations are subject to the usual rules of statistical inference.

References

1. RC Thomas, JA Coles, JW Deitmer: **Homeostatic muffling**. *Nature* 1991, **350:** 564.
2. CJ Schwiening, RC Thomas: **Relationship between intracellular calcium and its muffling measured by calcium iontophoresis in snail neurones.** *J Physiol* 1996, **491 (Pt 3):** 621-633.
3. E Neher: **The use of fura-2 for estimating Ca buffers and Ca fluxes.** *Neuropharmacology* 1995, **34:** 1423-1442.
4. PJ Bauer: **The local Ca concentration profile in the vicinity of a Ca channel.** *Cell Biochem Biophys* 2001, **35:** 49-61.
5. BK Vanselow, BU Keller: **Calcium dynamics and buffering in oculomotor neurones from mouse that are particularly resistant during amyotrophic lateral sclerosis (ALS)-related motoneurone disease.** *J Physiol* 2001, **525:** 433-445.
