# Supplementary material for: The quantitation of buffering action II. Applications of the formal & general approach
Source: Theor Biol Med Model. 2005 Mar 16;2:9. doi: 10.1186/1742-4682-2-9 (PMC1079954; doi:10.1186/1742-4682-2-9)
Supplement: Additional File 6 — Buffering and Muffling in Systems and Control Theory [file 1742-4682-2-9-S6.pdf]

Theoretical Biology and Medical Modelling

Research

The quantitation of buffering action. II. *Applications of the formal and general approach*.

Bernhard M. Schmitt

Supplement 6:

Buffering and Muffling in Systems and Control Theory

The concepts of buffering and "muffling" are closely related to the paradigms of signal processing and of systems and control theory. In the previous sections, we established a framework that could handle buffered systems even when they were dimensionally heterogeneous (e.g. autoregulation of flow in the face of pressure variations) or time dependent (e.g. the "muffling" of calcium). Control systems can be viewed as systems that are both, i.e., dimensionally heterogeneous and time dependent. By combining the two approaches established above, our buffering concept can be extended to systems and control theory. This extension will prove fruitful inasmuch it allows one to derive *1)* two novel quantitative measures of "systems level buffering" in time-independent, stationary systems, and *2)* two novel measures of control efficiency in time-dependent, non-stationary systems. More generally, this extension allows one to accommodate buffering and muffling within the language of control theory; as homeostatic mechanisms, this is where they belong.

Buffering: Describing the steady-state behavior of a system using the formal and general buffering concept

Buffered systems as descriptions of the stationary states of a system

Consider a generic buffered system

$$B=\{y=\tau(x),\ z=\beta(x)\},$$

which is, in principle, complete and consistent in itself as a purely mathematical idea. We can associate, however, specific meanings with its variables and parameters without altering the system's mathematical properties, and we have successfully done so repeatedly in this article. Now, we interpret the xyz-triplets of a buffered system as the stationary states of a feedback control system with three characteristic variables. For instance, a system with a fixed setpoint $R=0$ can be described in terms of the system variables "error" or "disturbance" input D, and two output variables Y and Z. Taking the disturbance input as the independent variable, we can write the "stationary

state space" of the control system as

$$\begin{pmatrix} D \\ Y(D) \\ Z(D) \end{pmatrix} \leftrightarrow \begin{pmatrix} x \\ y(x) \\ z(x) \end{pmatrix} = \begin{pmatrix} x \\ \tau(x) \\ \beta(x) \end{pmatrix},$$

or simply as $B\{\tau(x), \beta(x)\}$. Such an ordered combination of two functions $\tau(x)$ and $\beta(x)$ comprises all the information that is needed to derive the "static control properties" of this single input – double output system.

Recall that we have formulated above a classical buffering process (H^+ buffering by weak acids) in terms of a stationary state space. In that case, the state space represented all possible equilibrium states of a chemical "system" with three interdependent quantities:

$$\begin{pmatrix} [H^+]_{total} \\ [H^+]_{free} \\ [H^+]_{bound} \end{pmatrix} \leftrightarrow \begin{pmatrix} x \\ y \\ z \end{pmatrix} = \begin{pmatrix} x \\ \tau(x) \\ \beta(x) \end{pmatrix}.$$

Albeit chemists and control engineers use different vocabularies to describe the particular stationary systems they are studying, the respective quantitative descriptions share the same mathematical structure. Consequently, we may also merge the respective repertoires of mathematical tools and measures that are available in any of these disciplines for the analysis of such systems. In some cases, one merely needs to realize the correspondance between existing measures. In other cases, a measure may exist in one discipline, but lack in another; then, it that measure needs to be "translated" into the other terminology. Such transfers may occur in either direction and can enrich both the paradigm of buffering and that of control.

In a previous section, we already borrowed from signal processing theory the concept and graphical representation of "transfer elements" in order to describe the quantitative aspects of blood pressure buffering. Herein, the signal processing term "transfer factor" or "transfer coefficient" was found to become perfectly equivalent under certain conditions to the term "transfer coefficient" as used in our buffering concept. Similarly, one might employ other signal processing measures in order to describe buffering processes and "buffered systems". Examples include the "damping coefficient D":

$$D = \frac{\text{input}}{\text{output}} \leftrightarrow \frac{x}{y};$$

the "attenuation A", i.e., the logarithmic expression of the damping coefficient given in deciBel:

$$A = 10 \times \log \frac{\text{input}}{\text{output}} \text{dB} \leftrightarrow 10 \times \log \frac{x}{y};$$

or the "gain G", i.e., the logarithmic measure of amplification expressed in deciBel:

$$G = 10 \times \log \frac{\text{ouput}}{\text{input}} \text{dB} \leftrightarrow 10 \times \log \frac{y}{x}.$$

In contrast to our four buffering parameters which are genuine differentials, these units rely on the ratio of absolute values. For nonlinear systems, these parameters will therefore yield averages rather than specific indices of the local system behavior at a single point.

Reversly, one can apply the categories and parameters of our buffering concept to describe the properties of transfer elements; one example was our description of blood pressure buffering.

"Sensitivities" or "control coefficients" are equivalent to transfer and buffering coefficient of conservative systems.

One example for a measure that already exists in both the "buffering" and the "systems and control" paradigm, albeit under different names, is "sensitivity". "Sensitivity analysis", popular in natural and social sciences such as enzymology or econometrics, utilizes "sensitivity" as a measure for how strongly a given system parameter impacts on the overall state of the system. Specifically, sensitivity is defined as the partial derivative of one state variable with respect to a given system parameter. Usually, sensitivity analysis is restricted to the time-independent, steady states. For instance, shifting the disturbance input D in a single input-dual output control system by a certain amount ΔD to a new value will, after the initial transients have settled, permanently shift the steady-state outputs Y

and Z by respective amounts of ΔY and ΔZ. The respective sensitivities of output Y and output Z are

$$S_Y = \frac{dY}{dD} \leftrightarrow \frac{dy}{dx} = \tau'(x),$$

and

$$S_Z = \frac{dZ}{dD} \leftrightarrow \frac{dz}{dx} = \beta'(x).$$

Thus, the terms "sensitivity of output Y with respect to disturbance D" and "sensitivity of output Z with respect to D" in systems and control terminology are identical with the first derivative of the transfer function and buffering function, respectively, as used in the context of buffered systems. Derivatives and "sensitivities" may be either dimensionless numbers (in dimensionally homogeneous systems) or of a certain physical dimension (in dimensionally heterogeneous systems). In the special case of conservative buffered systems, the derivatives τ'(x) and β'(x) become equal to the transfer coefficient t(x) and the buffering coefficient b(x), respectively. In any type of system, the two sensitivities of a single-input dual-output system allow to compute all four buffering parameters t, b, T, and B *(see next paragraph)*.

Elasticities

A related term is "elasticity" or "relative sensitivity". Just like sensitivity, it measures the impact of a parameter change on a system output, but is computed from the relative changes. For instance, the elasticity of output Z with respect to the disturbance D in this system is

$$E_Z = \frac{dZ/Z}{dD/D} = \frac{d\ln(Z)}{d\ln(D)}.$$

Years before this concept of "elasticitiy" was introduced by engineers, a formally equivalent measure was used by physiologists in order to quantitate the autoregulation of renal blood flow in the face of variable blood pressure as

$$\frac{\Delta(\text{blood flow})/(\text{blood flow})}{\Delta BP/BP} = \frac{d\ln(\text{blood flow})}{d\ln(\text{blood pressure})} \text{ [1].}$$

"Elasticities" are furthermore used in enzymology to express the relative sensitivity of an enzyme to substrate concentration. Relative sensitivies are also popular in the analysis of entire metabolic networks; when used in this context, relative sensitivities are often called "control coefficients". For instance, the effect of enzyme activity changes on steady-state concentration and flux of a given metabolite are termed "concentration control coefficient" and "flux control coefficient", respectively .

If desired, "elasticities" can be translated into our concept of buffering as

$$E_y = \frac{d\ln(y)}{d\ln(x)}$$

and

$$E_z = \frac{d\ln(z)}{d\ln(x)}.$$

Elasticities are always dimensionless numbers, similar to the four buffering parameters t, b, T, and B. On the other hand, elasticities suffer from the usual disadvantages of logarithmic units, including the inability to express zero or negative values, and sensitivity to y- or z-offsets at x=0. In contrast, the four buffering parameters are equally dimensionless, but not subject to the limitations mentioned. To our knowledge, the equivalents to the four buffering parameters t, b, T, and B have not been introduced in systems and control theory. It is straightforward, however, to do so.

Computing the four buffering parameters t, b, T, and B in control systems

As shown above, in a dimensionally heterogeneous control system with a single disturbance input D, two outputs Y(D) and Z(D), the following correspondences exist between buffered systems and control systems *(Figure 1A)*:

$$\tau'(x) \leftrightarrow S_Y(D)$$

$$\beta'(x) \leftrightarrow S_Z(D)$$

$$\sigma'(x) = \tau'(x) + \beta'(x) \leftrightarrow K(D) = S_Y(D) + S_Z(D).$$

The conversion factor K(D) characterizes the "unbuffered" system response and may vary as a function of the disturbance input D. In the context of a feedback control system, "unbuffered response" is tantamount to "open loop sensitivity" of Y to D, i.e., to the sensitivity $S_Y(D)$ that is observed with the

Figure 1: Measures of control performance in control systems.

A-C, Feedback control system with proportional negative feedback.

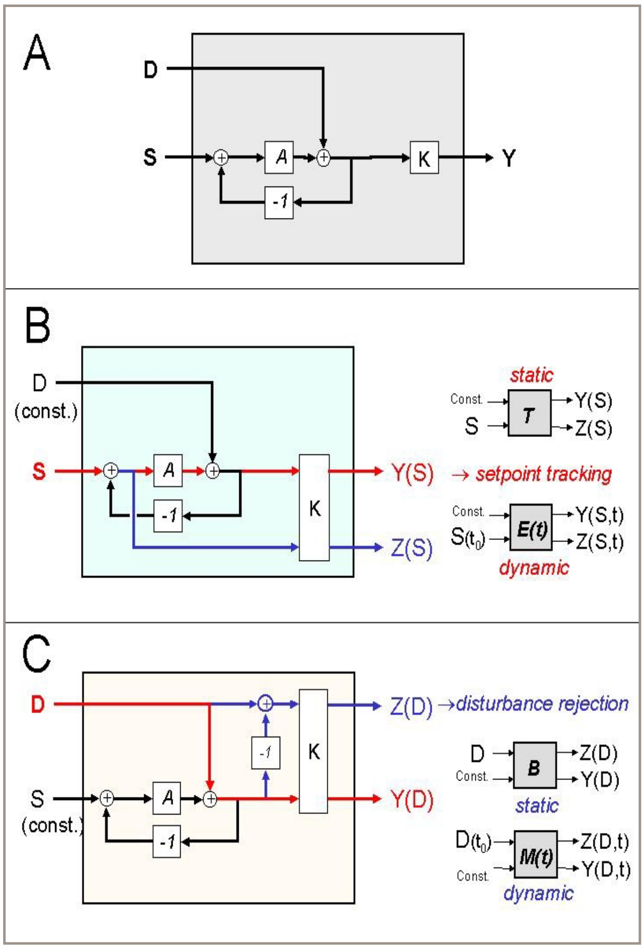

A, Principle.

The system transduces a setpoint S into an output Y of same or different physical dimension as S, reflected in a proportionality factor K. Control in the face of a disturbance input D is enabled by negative feedback. Control performance may be assessed either in terms of "setpoint tracking" or of "disturbance rejection" in the face of a disturbance input D.

B, Quantifying "setpoint tracking" in a proportional feedback control system.

Perfect control in the sense of perfect translation of the setpoint S into an output Y(S) is defined by Y(S)=K×S in the steady state. With less than perfect translation, Y(S) is only a fraction of K×S. For a fixed disturbance input D (or "load") of the system, the „transfer ratio" T(S) provides a measure of "static setpoint tracking power". The transfer ratio is computed as dY(S)/d[K×S-Y(S)]. The quantity [K×S-Y(S)], reflecting the attenuation of „buffering" of the input S, is an internal system variable that can be read out as a second output Z(S) (bold arrows). A measure of „dynamic setpoint tracking" can be computed from the integrated time courses of the two outputs.

C, Quantifying"disturbance rejection" in a proportional feedback control system.

Without any disturbance rejection, a given disturbance ΔD translates completely into a change of output Y of amount ΔD×K. With some disturbance rejection, the change of Y(S) will be only a fraction of ΔD×K. For a fixed setpoint input S, the „buffering ratio" B(D) provide a measure of „static disturbance rejection power". The disturbance rejection ratio is computed as dY(D)/d[K×D-Y(D)].

The quantity [K×D-Y(D)], corresponding to the attenuation of „buffering" of the disturbance input, is an internal system variable that can be represented as a second output Z(D) of the system (bold arrows). A measure of „dynamic disturbance rejection" can be computed from the integrated time courses of the two outputs.

If either the disturbance or the setpoint input are kept constant, the measures of static and dynamic control performance can be computed in a „black box" approach in any type of control system, independent from the particular control protocol employed. T, transfer ratio, a measure of static setpoint tracking; B, buffering ratio, a measure of static disturbance rejection; E(t), dynamic setpoint tracking ratio; M(t), dynamic disturbance rejection ratio, equivalent to the „muffling ratio".

feed-back limb disrupted. With these correspondances, it is straightforward to express the four buffering parameters in terms of the relevant control system quantities:

Transfer coefficient: $t(D)=\frac{S_Y(D)}{K(D)}$

Buffering coefficient: $b(D)=\frac{S_Z(D)}{K(D)}$

Transfer ratio: $T(D)=\frac{S_Y(D)}{S_Z(D)}=\frac{S_Y(D)}{K(D)-S_Y(D)}$

Buffering ratio: $B(D)=\frac{S_Z(D)}{S_Y(D)}=\frac{S_Z(D)}{K(D)-S_Z(D)}$.

Taken individually, none of the three basic quantities [$S_Y(D)$, $S_Z(D)$, $K(D)$] implies any information about "buffering". In contrast, the four secondary measures t, b, T, and B, "imported" from our buffering concept, provide the key to the stringent definition and quantitation of buffering in the context of systems and control theory. In particular, the buffering ratio B allows one to quantitate "systems level buffering strength" with respect to an external disturbance by means of a genuine ratio scale.

Given the explicit and axiomatic form of our general and formal buffering concept *(Buffering I)*, this extension onto systems and control theory appears obvious. Nonetheless, the result of this minor stretch is unique and valuable inasmuch current control theory holds no explicit, rigorous unit for the quantitation of systems level buffering, and the term "buffering" has remained vague or metaphorical in this specific context.

As a special case of general buffering, "systems level buffering" shares all formal characteristics and requirements of the former. Thus, the four measures t, b, T, and B are dimensionless, and T and B yield scales with an absolute zero and equal intervals, i.e., genuine ratio scales.

The open loop-response must be known in order to quantitate systems level buffering

The explicit form of the buffering parameters makes it obvious what minimal information is required for the quantitation of buffering action in control systems: One may determine the response of one output (Y or Z) plus the open loop-response of output Y (i.e., the converson factor K) and compute the buffering ratio as:

$$B(D)=\frac{S_Z(D)}{K(D)-S_Z(D)}.$$

Alternatively, one may measure simultaneously the response of both outputs Y and Z, yielding the buffering ratio as:

$$B(D)=\frac{S_Z(D)}{S_Y(D)}.$$

The open loop response is thus known in both cases, either explicitly as the conversion factor $K(D)$, or implicitly as the sum S_Y+S_Z of the two sensitivities.

By the same token, if one is unable to tell how the system would behave with zero buffering, then one cannot quantitate buffering at all. This criterion allows one to distinguish whether the buffering paradigm is applicable or not in a particular situation. For instance, we concluded above that "blood pressure variability buffering" cannot be described in terms of buffering because we could not find an explicit description of blood pressure variability for zero buffering *(Buffering II - Supplement 4)*.

Furthermore, the two roles of transfer and buffering function must be assigned explicitly and unambiguously to the two outputs. In order to form a dual-output system that conforms to the framework for two-partitioned and buffered systems, multiple outputs-systems must be transformed into a two-output system, and systems with a single "physical" output need to be complemented with the second output using the conversion factor K:

$$Y(D)=K(D)-Z(D)$$

or

$$Z(D)=K(D)-Y(D).$$

These processes were termed $^n\Pi\rightarrow{}^2\Pi$ transformations and detailed in the accompanying paper *(Buffering I – Supplement 8)*. Physical dimensions of input variable vs. both output variables may differ (i.e., "dimensionally heterogeneous systems"), but the two output

variables Y and Z must be of identical physical dimension.

The buffering ratio B provides an absolute ratio scale for "stationary disturbance rejection" in control systems

A thermostat as buffered system

What does "buffering" mean, and what is measured by the buffering ratio B in the context of control systems? Consider a simple feedback control system such as a thermostatted water bath. Initially, water bath and ambient air shall have the same, constant temperature.

Then, ambient temperature is increased by a certain amount, corresponding to a step change ΔD of the disturbance input D. The resulting heat flow from ambient air into the water bath tends to increase bath temperature until a new steady state level is attained.

We interpret bath temperature as the output Y, and the change of bath temperature as ΔY. The particular relation between disturbance ΔD and bath temperature change ΔY depends on the properties of the thermostat.

Computing the buffering ratio B of a feed-back control system

In one limiting case, the thermostat is switched off and bath temperature will ultimately follow ambient temperature completely, such that ΔY=ΔD. In this case, there is apparently no buffering of the output Y with respect to the disturbance D. To see how this is reflected in the buffering ratio B, one needs to know, in addition to ΔY, either the change ΔZ of the second output, or the conversion factor K(D).

Clearly, the system's behavior represents the open loop response. The conversion factor therefore follows as K(D)=1, and the open loop response as ΔY=K×ΔD=1×ΔD. A conversion factor K=1 was shown to be the hallmark of "conservative systems". We can thus compute ΔZ as ΔZ = ΔD-ΔY = 0, and hence the buffering ratio B as $B=\frac{dZ}{dY}=0$. A buffering ratio of zero appropriatey reflects the absence of buffering observed when the controller is switched off.

Next, assume that the thermostat is switched on and works very efficiently. Then, the coolant running inside the coils extracts exactly as much heat from the water as is flowing in from the ambient. Consequently, bath temperature in the new steady state is unchanged as compared to the initial steady state, such that ΔY=0.

Intuitively, one would say in this case that the buffering of the output Y against ΔD is perfect. Fittingly, if ΔY approaches 0, we find that the buffering ratio

$$B=\frac{dZ}{dY}$$

approaches infinity.

A positive buffering ratio of intermediate magnitude is found at high ambient temperatures when heat inflow becomes greater than the maximum outflow supported by the thermostat. Water temperature will then rise, though to a lesser extent than ambient temperature (0<ΔY<ΔD).

"Disturbance rejection" as a virtual quantity

In all these examples, the variable Y simply indicated the water temperature, and ΔY the change produced by the incremental disturbance ΔD. In contrast, neither Z nor ΔZ have any direct physical equivalent. However, the "virtual" quantity ΔZ may be thought of as that portion of the temperature change ΔD that was diverted from the water, or "rejected".

"Disturbance rejection", i.e., the ability to preserve the system state in the face of an external disturbance, is one fundamental property of control systems. The second fundamental property termed "setpoint tracking" may be quantitated conveniently with the aid of the "transfer ratio " T *(see following paragraph)*.

Buffering coefficient b(D) and buffering ratio B(D) provide a quantitative measure of "disturbance rejection" at a given state of the system. One might call these measures "disturbance rejection coefficient" and "disturbance rejection ratio", respectively.

Reconsider the thermostatted water bath. On the physical level, its behavior is characterized

completely by the relationship between only two physical variables, namely ambient temperature ϑ_X and water temperature ϑ_Y. These two are not sufficient, however, to speak meaningfully of "disturbance rejection". In addition, one must obviously know the magnitude of the disturbance that was rejected. This distinction was made possible by the assumption that $\Delta Y+\Delta Z=\Delta D$ which yielded the "virtual" second output ϑ_Z, the "rejected" temperature.

In a similar fashion, one may construct the "virtual" second output Z for other control systems; examples are the "buffering" of body temperature against changes of ambient temperature, the "buffering" of cytoplasmic concentrations of K^+, Na^+, H^+, Ca^{++} etc. against changes of the respective extracellular concentrations, or the buffering of renal perfusion against changes of cardiac output.

"Disturbance rejection" equals "resistance to change"

Recall that many textbooks define chemical buffering as "resistance to change" with respect to pH. The similarity between the semantics of "disturbance rejection" and "resistance to change" provides an intuitive, conceptual bridge between the two paradigms of buffering and control. The similarity is not merely metaphorical. Rather, with the dimensionless buffering ratio B we are in a position to take "resistance to change" literally in its general meaning and quantitate the resistance to all kinds of change, not just to changes in pH or pCa.

By hindsight, it appears odd to think that "resistance to change" should be something that is expressed in terms of "millimoles per liter".

The transfer ratio T provides an absolute ratio scale for stationary setpoint tracking in control systems

Besides disturbance rejection, a second crucial ability of control systems is "setpoint tracking", i.e., the ability to move the system quickly and completely from one state to another.

Setpoint tracking in a thermostatted water bath

For a thermostatted water bath, this means the ability to adjust water temperature quickly and exactly when a new desired temperature is set on the dial. Limiting our analysis to stationary states, the system is again fully characterized by one input variable (i.e., the setpoint S) and two outputs as above (i.e., water temperature Y and "rejected input" Z). Note, however, that the meaning of the input variable now changed from an external disturbance D to a setpoint S. The external disturbance D is assumed to be fixed at $D=0$, whereas the setpoint S is allowed to vary. Input S and both outputs Y and Z have the dimension of a temperature.

With the input now being a setpoint rather than a disturbance, a well-designed system is expected not to reject that input, but to translate it faithfully into corresponding changes of Y. Real systems may achieve perfect translation of ΔS such that $\Delta Y=\Delta S$, e.g. by employing integral feed-back strategies. Often enough, control is not perfect, and a steady-state error remains, e.g. with solely proportional feed-back.

Computing the transfer ratio T for a feed-back control system

The efficiency of this translation process can be expressed conveniently and rigorously using the transfer coefficient t(S) or the transfer ratio T(S), computed as

$$t(S) = \frac{S_Y(S)}{K(S)}$$

and

$$T(S) = \frac{S_Y(S)}{S_Z(S)}.$$

The transfer ratio T provides a ratio scale. These measures of setpoint tracking may be termed "setpoint tracking coefficient" and "setpoint tracking ratio ", respectively.

Robustness and fragility in control systems

"Robustness" and its complement "fragility" are popular terms in systems and control theory, but there is no clarity as to how these properties could be quantified.

As a measure of fragility, Csete & Doyle suggested the logarithm of the absolute sensitivity $S_Y(D)$ of an output Y to a disturbance input D:

$$fragility = \log|S_Y(D)|,$$

where $S_Y(D)=dY(D)/dD$. Clearly, however, taking the logarithm of the sensitivity S_Y does not create a novel, independent quantity. Rather, the result is reduced information content (the sign is lost by using the absolute of S_Y) and reduced versatility (zero sensitivities cannot be represented). Furthermore, if sensitivites are not dimensionless numbers, the corresponding logarithms are not defined; transformation into a dimensionless quantity, on the other hand, critically determines the measure of fragility, yet is an arbitrary, unstandardized step. Similar criticisms apply to robustness, which was defined as *"roughly the inverse of fragility"* [2], yielding:

$$robustness = 1/\log|S_Y(D)|.$$

In contrast, our buffering ratio (or static disturbance rejection ratio) $B(D)$ can provide a measure of robustness that carries additional information not implied by the sensitivity S_D. Moreover, the buffering ratio is always dimensionless and normalized with respect to the open loop sensitivity of the system. Similarly, the transfer ratio $T(D)$ can provide a rigorous measure of "fragility".

Muffling: Describing the time-dependent behavior of a system using the formal and general buffering concept

The muffling ratio M(t) provides an absolute ratio scale for dynamic disturbance rejection in control systems

Above, we introduced the integrals $\varepsilon(t)$ and $\mu(t)$ as time-weighted measures of additional free and muffled Ca^{++} ions, respectively, in response to an acute Ca^{++} load. Furthermore, we used their ratio $\mu(t)/\varepsilon(t)$, i.e., the muffling ratio $M(t)$, for the quantitation of muffling. "Muffling" of Ca^{++} ions is one prominent example of *time-dependent* disturbance rejection. The quantitative measure of muffling [the muffling ratio $M(t)$] can be applied as well in the context of a classical feedback system.

Time-dependent disturbance rejection by a voltage clamp device

Consider, for instance, a voltage-clamp device that is used to control the membrane potential of a cell. We interpret the fixed command potential V_{com} as a fixed set-point S, and the actual membrane potential V_m as output Y. Initially, membrane resistance shall be constant and well controlled ($V_m=V_{com}$ and $Y=S$). One task of the clamp device then is to keep V_m constant in the face of external disturbances such as fluctuations of membrane current I_m. To challenge the device in a defined way, we may impose such a disturbance ΔD by continuously injecting a known additional current ΔI_m.

This maneuver will produce a voltage step $\Delta V_m=\Delta I_m \cdot R_m$. Without control, e.g. when the clamp device is switched off, the deviation ΔV_m will persist at this constant new value however long one waits, such that $\Delta V_m(t)= \Delta V_m(0)$. The time integral $\varepsilon(t)$ of the deviation $e(t)$ is then given by

$$\varepsilon(t) = \int_0^t \Delta V_m(t)\cdot dt = \Delta V_m \times t,$$

and the time integral $\mu(t)$ of the "muffled" disturbance $m(t)$ as:

$$\mu(t) = \int_0^t \left(\Delta V_m(0) - \Delta V_m(t)\right)\cdot dt = 0.$$

The muffling ratio $M(t)$ follows as

$$M(t) = \frac{\mu(t)}{\varepsilon(t)} = \frac{0}{\Delta V_m \times t} = 0.$$

The muffling ratio as a measure of control speed and efficiency will thus appropriately indicate "zero control" for all values of t.

With good feed-back control, on the other hand, the initial voltage deflection $\Delta V_m(t)$ will travel towards zero over time. "Perfect control" would mean that the disturbance is compensated completely and instantaneously such that $\Delta V_m(t)=0$ for all $t>0$. We then find for the error integral that $\varepsilon(t)=0$, and for the integrated muffling function that

$$\mu(t) = \int_0^t \left(\Delta V_m(0) - \Delta V_m(t)\right) \cdot dt = \Delta V_m(0) \times t \,.$$

From this, the muffling ratio follows as

$$M(t) = \frac{\Delta V_m \times t}{0} \leftrightarrow \infty \,.$$

An "infinite muffling ratio" again reflects appropriately the presence of "perfect control".

The control efficiency of "real world" clamp devices lies somewhere in between zero and perfect control. Control quality in the sense of time-weighted disturbance rejection depends on both speed and fidelity: *1)* the faster $\Delta V_m(t)$ returns to zero, the better the control quality, and *2)* the closer $\Delta V_m(t)$ returns to the baseline, the better the control quality. Herein, the relative importance of speed decreases with longer time windows, and the importance of clamp fidelity increases.

The actual path of $\Delta V_m(t)$ over time may take very diverse shapes, depending on the design and tuning of the controller (e.g. proportional-differential-integral feed-back, gain and time constants, or analog vs. digital control). Whatever the inividual response of a given controller may look like, it is always possible to extract the functions $\varepsilon(t)$ and $\mu(t)$ from the observed trajectory of $\Delta V_m(t)$ together with the known disturbance ΔI_m, and thus derive the muffling ratio $M(t)$ as an overall measure of disturbance rejection efficiency.

For reasons of clarity, we can call the measure $M(t)$ in this context also "dynamic disturbance rejection ratio ".

The dynamic transfer ratio E(t) provides a ratio scale for dynamic setpoint tracking in control systems

Time-dependent setpoint tracking by a voltage clamp device

The voltage clamp device can be challenged in another way. Namely, while holding the external disturbance at a constant value D_0, the command potential may be stepped up instantaneously by an amount ΔV_{com}. Without control, this maneuver will not elicit any response at all, and membrane potential will persist at the baseline value ($\Delta V_m = 0$).

Then, the time intgral of the voltage deviation from the initial value is

$$\varepsilon(t) = \int_0^t \Delta V_m(t) \cdot dt = 0 \;\;.$$

In this context, $\varepsilon(t)$ represents a desired excursion from the initial value rather than an undesired "error". Furthermore, the time integral of the "muffled" portion of the setpoint change is

$$\mu(t) = \int_0^t \left(\Delta V_{com} - \Delta V_m(t)\right) \cdot dt = \Delta V_{com} \times t \,.$$

Muffling of the setpoint change is not desired in this context; rather, $\mu(t)$ takes the meaning of an unwanted clamp error integrated over time. Without control, we find a muffling ratio of

$$M(t) = (\Delta V_{com} \times t)/(0 \times t) \leftrightarrow \infty \,.$$

In words, suppression (or muffling) of the extra command was perfect. We now define a "dynamic transfer ratio" with respect to a given step change ΔS of the setpoint S:

$$E(t) \equiv \frac{\varepsilon(t)}{\mu(t)} \,.$$

The dynamic transfer ratio provides a measure of clamp efficiency in the sense of setpoint tracking; they might be called "dynamic setpoint tracking ratio" as well. In our example, their value equals zero: $E(t) = (0 \times t)/(\Delta V_m \times t) = 0$. Translation of the extra command was zero.

In contrast, an ideal controller would be capable to switch V_m instantaneously and completely to the new voltage $V_m = V_{com} + \Delta V_{com}$. From the dynamic transfer function

$$\varepsilon(t) = \int_0^t \Delta V_m(t) \cdot dt = \Delta V_{com} \times t$$

and the muffling function

$$\mu(t) = \int_0^t \left(\Delta V_{com} - \Delta V_m(t)\right) \cdot dt = 0 \,,$$

we calculate the dynamic setpoint tracking ratio as

$$E(t) = \Delta V_{com}(t)/0 \leftrightarrow \infty.$$

"Infinite" setpoint tracking ratio adequately represents the underlying perfect control.

Real world controllers will again track the setpoint with finite speed and precision, and with a

potentially complex time course of $\Delta V_m(t)$. It is always possible to determine from the time course of $V_m(t)$ the parameters $\varepsilon(t)$, $\mu(t)$, and the dynamic setpoint tracking ratio $E(t)$ as a time-weighted measure of setpoint tracking efficiency.

Dimensions of control quality that are represented by the static and dynamic measures of setpoint tracking and disturbance rejection

Systems and control theory offers several measures of control quality, such as steady-state error, overshoot, settling time, integral of absolute error (IAE), integral of squared error (ISE), integral of time-multiplied absolute error (ITAE) etc.. Clearly, it is impossible to condense all potentially relevant aspects of control into a single figure.

The four absolute ratio scales derived from our general buffering concept [stationary setpoint tracking ratio $T(S)$, stationary disturbance rejection ratio $B(D)$, dynamic setpoint tracking ratio $E(t)$, dynamic disturbance rejection ratio $M(t)$] allow to quantitate particular aspects of control efficiency in a novel way.

We discuss these aspects in terms of the four fundamental dimensions of control efficiency: system stability, system accuracy in the stationary state, damping of the transient response, and speed of the transient response.

Buffering and the stability of control systems

According to the "BIBO" criterion, a system is stable if a bounded input results in a bounded output. "Bounded output" may mean a stationary state where the output is a constant value (e.g. body temperature or cruising speed), but a bounded output may also manifest as oscillations around a constant value (e.g. blood pressure or corticosterone plasma levels).

Our original definitions of the stationary measures "setpoint tracking ratio" $T(S)$ and "disturbance rejection ratio" $B(D)$ rest on the ratios $dZ(D)/dY(D)$ and $dY(S)/dZ(S)$, respectively, of two time-independent outputs Y and Z. These measures can thus be computed only if the system is BIBO stable *without* oscillations. If oscillations are present, e.g. as for blood pressure, the outputs do not approach a fixed value with time, and the corresponding ratios cannot be computed. Then, one may resort to an alternative definition in order to compute these two measures.

As shown above in the section about "muffling", buffering can be considered as the limiting case of muffling for infinitely small disturbances an infinitely long integration time. As long as the amplitude of the oscillations does not increase with time, the limit of the dynamic disturbance rejection ratio

$$M(t,D,\Delta D) = \frac{\int_0^t m(t)\cdot dt}{\int_0^t e(t)\cdot dt} \text{ for } (\Delta D \to 0) \text{ and } (t \to \infty)$$

approaches a fixed value that is equal to the static disturbance rejection ratio $B(D)$. In a similar way, we can compute the dynamic setpoint tracking ratio even in the presence of oscillations as the limit of

$$E(t,D,\Delta D) = \frac{\int_0^t e(t)\cdot dt}{\int_0^t m(t)\cdot dt} \text{ for } \Delta S \to 0 \text{ and } t \to \infty.$$

Thus, the static disturbance rejection and setpoint tracking ratio can be computed for all BIBO-stable systems.

An interesting application of these two measures of static control efficiency are oscillators. Certain BIBO-stable systems with a stationary output are suited as building blocks for higher order systems that can behave as oscillators [3]. For instance, electrical resistors with a negative slope conductance dI/dV (implying also negative resistance dV/dI) can be combined with an energy source (such as a battery) and an energy storage device (such as a capacitor) to form an electrical oscillator. Heartbeat, sleep, reproductive cycles, signal transduction and numerous further physiological processes are controlled by electrical or other biological oscillators.

Oscillators play important roles in other disciplines as well. An example related to chemical buffering are pH oscillators [4]. For these systems, criteria equivalent to the electrical one of "negative slope conductance" are required. Our buffering concept provides such a formal criterion in a simple and general form: A buffered system can be used to build an oscillator if it exhibits "inversion", i.e., if

the transfer ratio t (equal to the static setpoint tracking ratio) is negative, including inversion-moderation and inversion-amplification *(Buffering I)*.

In the absence of BIBO-stability, the dynamic setpoint tracking ratio E(t) or dynamic disturbance rejection ratio M(t) can be computed as discussed, and provide useful information. These measures can be applied even of "catastrophic" processes, characterized by outputs that ultimately approach $+\infty$ or $-\infty$.

In either case, both measures of dynamic control performance will approach more or less quickly a characteristic limiting value of –1. Up to any given finite time t, however, unstable systems clearly differ quantitatively among each other with respect to some limited, temporary control they may provide, or to the harm that they may cause.

Control performance of these systems can be assessed and compared using the parameters E(t) and M(t) as originally defined: At a given time t, dynamic setpoint tracking is better for the system with the greater dynamic setpoint tracking ratio E(t), and dynamic disturbance rejection is better for the system with the greater dynamic disturbance rejection ratio M(t).

Buffering and stationary precision

The stationary setpoint tracking ratio T(S) and the static disturbance rejection ratio B(D) provide direct measures of stationary precision in the respective contexts of setpoint changes or external disturbances. Herein, T(S) and B(D) yield scales of the highest possible type, namely absolute ratio scales.

The fact that these measures of controller performance are dimensionless represents a great advantage as compared to simply reporting the steady state error in the physical unit of the regulated variable: A single, generally applicable unit allows one to compare the static control performances of systems that regulate rather diverse parameters.

The fact that these measures of controller performance yield values on a ratio scale have the advantage that one can meaningfully indicate by what factor control quality in one system differs from that of another; the absolute character of the scales endowes the measures with an absolute and unambiguous meaning, without the need to define additional unit or scaling conventions.

Buffering and damping of the feed-back response

It is desirable for the control of many parameters that the response to changing disturbance D or setpoint S produces only short-lasting transients before attaining a new steady-state, as opposed to persistent oscillations. For instance, damping is of paramount importance for the regulation of a watch-maker's finger movements, or for the regulation of cruise altitude during the landing of an aircraft.

In other situations, however, a certain degree of damping is irrelevant with respect to other specific control goals. For instance, the cyclical pH-swings associated with breathing are negligible for acid-base balance; similarly, it does not matter for the quality of a lawn whether the appropriate volume of water is rained on by a device that oscillates back and forth or by continuous irrigation at a fixed rate.

Finally, damping can be outright counterproductive in certain situations: In the blood circulation, for instance, the cyclic pressure changes help to return venous blood to the heart, and perfusion at a fixed pressure would negatively affect control quality. Similarly, maximizing damping would clearly not improve control performance when trying to control the position of a plate while juggling it on top of a stick.

Because no strictly stationary state is ever attained in undamped systems, our measures of stationary control performance, T(S) and B(D), cannot be computed directly as dy/dz and dz/dy, respectively, but as the respective dynamic measures E(t) and M(t) for the limiting case of $\Delta S \to 0 / \Delta D \to 0$ and $t \to \infty$. Then, these two dynamic measures are insensitive to symmetric oscillations.

In this sense, E(t) and M(t) describe the efficiency with which the average of a certain parameter (rather than the parameter itself) is controlled within the given time window. Reversly, these measures do not contain any information about the possible damping of that parameter.

In conclusion, the measures T(S) and B(D) of stationary control efficiency are useful for processes in which oscillations are either required for the intended purpose of the control system or may be ignored, e.g. because oscillation frequency is too high, or amplitude too small.

Buffering and the speed of the feed-back response

By definition, the two measures of static control efficiency, T(S) and B(D), depend exclusively on steady-states (or the average value around which a variable may oscillate). Thus, T(S) and B(D) are completely insensitive to the speed of the feed-back response which exclusively impacts on the pre-steady state. On the other hand, this implies that T(S) and B(D) can be computed only in BIBO-stable systems.

The two measures of dynamic control efficiency, E(t) and M(t), are determined by the combined effects of both control speed and control precision, i.e., by the pre-steady state trajectory as well as by the position of the steady-state. This is in keeping with the notion that these measures describe the control of the controlled parameter's average over the time window considered. Thus, the relative contribution of speed (including the effects of any potential lags) is large for short integration times, but fades away to zero for progressively longer (provided the system is stable). When time is sufficiently long, and changes of setpoint S or disturbance D are sufficiently small, then the two dynamic measures E(t) and M(t) approach the value of the corresponding measures of steady-state control efficiency T(S) and B(D), respectively.

Importantly, what is considered a criterion of "control quality" depends on the time interval during which a parameter needs to be controlled. For instance, to an electrophysiologist working with a voltage clamp device, speed is essential for recording the fast gating currents, whereas measuring steady-state currents calls for maximized clamp fidelity. Typically, one would find that a proportional-differential (PD) controller is faster and gives better results for the gating currents, whereas a proportional-integral (PI) controller performs better at the second task because of its higher clamp fidelity.

Such qualitative statements may be verified with the aid of the two measures of dynamic control efficiency: In the first case, the challenge to the clamp consists in a step of command voltage. Consequently, clamp performance of the two devices is computed as "dynamic setpoint tracking ratio" E(t). In the second case, the clamp is challenged by current fluctuations at a constant holding potential, making the "dynamic disturbance rejection ratio" the appropriate measure of clamp performance in this case. The units E(t) and M(t) are dimensionless and allow direct comparison of control quality, in spite of the fact that the two time intervals are different. Moreover, it is even possible to directly compare the quality of "dynamic disturbance rejection" to the quality of "dynamic setpoint tracking".

Thus, damping is not measured directly by any of these units [T(S), B(D), E(t), and M(t)]. Static control performance, expressed as T(S) or B(D), cannot be defined for BIBO-unstable systems for lack of a stationary state. Dynamic control performance, on the other hand, expressed as E(t) or M(t), is defined for stable and unstable systems alike and only sensitive to instability inasmuch speed or precision are compromised.

Taken together, static setpoint tracking ratio T(S) and static disturbance rejection ratio B(D) are measures exclusively of stationary precision, whereas dynamic setpoint tracking ratio E(t) and dynamic disturbance rejection ratio M(t) measure the combined effect of stationary precision and speed of the feedback control.

References

1. SJG Semple, HE De Wardener: **Effect of increased renal venous pressure on circulatory "autoregulation" of isolated kidney.** Circulation Research 1959, **7:** 643-648.
2. ME Csete, JC Doyle: **Reverse engineering of biological complexity.** Science 2002, **295:** 1664-1669.
3. TF Fischer Weiss: Cellular Biophysics. Cambridge, MA, USA: MIT Press; 1997.
4. GP Misra, RA Siegel: **Multipulse drug permeation across a membrane driven by a chemical pH-oscillator.** J Control Release 2002, **79:** 293-297.
