# Supplementary material for: The quantitation of buffering action II. Applications of the formal & general approach
Source: Theor Biol Med Model. 2005 Mar 16;2:9. doi: 10.1186/1742-4682-2-9 (PMC1079954; doi:10.1186/1742-4682-2-9)
Supplement: Additional File 1 — Further Notes on Langmuir Buffering [file 1742-4682-2-9-S1.pdf]

Theoretical Biology and Medical Modelling

Research

The quantitation of buffering action. II. *Applications of the formal and general approach.*

Bernhard M. Schmitt

Supplement 1:

Further Notes on Langmuir Buffering

Earlier similar approaches to Langmuir buffering

A result equivalent to the expression

$$B(y) = \frac{c \times d}{(d+y)^2}$$

was published 80 years ago by Van Slyke [1]; the problems associated with Van Slyke's presentation were discussed in the preceding article *(Buffering I - Supplement 10)*. In the context of Ca^{++} ion buffering, Neher recently presented the analogous relationship

$$\frac{[\mathrm{Buffer}]_{\mathrm{total}} \times K_d}{(K_d + [Ca^{++}]_{\mathrm{free}})^2}$$

as the correct analytical solution for the "Ca^{++} binding ratio κ_S" which he had defined as

$$\kappa_s = \frac{d[Ca^{++}]_{\mathrm{bound}}}{d[Ca^{++}]_{\mathrm{free}}}.$$

Thus, Neher's Ca^{++} binding ratio κ_S is the exact Ca^{++}-specific equivalent of the buffering ratio

$$B(y) = \frac{d(\mathrm{bound})}{d(\mathrm{free})} = \frac{dz}{dy}$$

in our general notation [2].

Physical model of Langmuir-type buffering

For positive constants c and d, the coefficients t and b will always assume values between 0 and 1. Such a buffering behavior, which is also characteristic of H^+ buffering in pure water *(Buffering II - Supplement 2)*, was classified as "non-inverting moderation" *(Buffering I)*. The accompanying paper presented a general method to construct communicating vessel-models of non-inverting moderation *(Buffering I - Supplement 4)*.

In order to visualize the buffering ratio B, we use a transfer vessel T that is a perfect cylinder with an area A_T at its base of A_T=1; then, the fluid volume y inside that vessel is numerically equivalent to the fluid level. The buffering vessel B then has a cross-sectional area of

$$A_B(y) = B(y) = \frac{c \cdot d}{(d+y)^2}.$$

This communicating vessels model can illustrate four characteristic system states of Langmuir buffers *(discussed in the main text)* which correspond to a system of empty vessels, with a buffering vessel half empty, with fluid volumes being similar in both

vessels, and with the cross-sectional areas at the fluid surface being similar in both vessels.

Comparison with other descriptions of H^+-buffering by weak acids.

The qualitative and quantitative conclusions regarding the buffering of H^+ ions by weak acids obtained here differ considerably from familiar textbook descriptions of weak acids and bases which are mostly based on Michaelis' and Van Slyke's unit β_{H^+} = dBase/dpH. Two discrepancies are particularly striking.

Position of maximum buffering strength

The unit β_{H^+} has a maximum at half-saturation of the buffer, i.e., at $pH=pK_A$ and $[H^+]_{free}=K_A$. On either side of that maximum, β_{H^+} decreases monotonically, approaching a value of zero as $[H^+]_{free}$ approaches either zero or infinity. In contrast, when one employs the measures b and B, buffering is maximal at $[H^+]_{free}=0$, and with increasing $[H^+]_{free}$ both b and B decrease monotonically towards zero.

From a mechanistical point of view, it appears evident that buffering efficiency should decrease if the number of available binding sites decreases. In contrast, accepting the notion that buffering with half the total binding sites occupied should be more efficient than buffering with all binding sites free and available for binding is counterintuitive and requires a considerable sacrifice of common sense.

Dependence of maximum buffering strength on binding affinity

At similar concentration, all monoprotic weak acids exhibit identical maximum buffering powers β_{H^+max} according to Van Slyke's unit. In particular, β_{H^+max} is independent of the individual pK_A values; the pK_A solely affects the position of the maxima. In contrast, maximum buffering according to the buffering coefficient b and the buffering ratio B is proportional to $1/K_A$ (i.e., the "affinity" of the buffer for the ligand) and thus decreases monotonically with increasing K_A. According to their individual "maximum buffering strengths", weak acids (small K_A, high pK_A) are better H^+ buffers than stronger acids (large K_A, low pK_A). Stated in yet another, equivalent way from a mechanistical point of view: A compound with a strong tendency to bind free H^+ ions makes a better buffer than a compound with a poor tendency to accept free H^+ ions. This latter statement is so simple and intuitive that it almost appears trivial - if it weren't for the deeply conflicting conclusions that are inevitably reached if one adopts the unit β_{H^+}.

Dependence of buffering strength on affinity at a fixed concentration of free ligand

Magnitude and position of the maximum buffering strength of a given buffer are thus very much dependent on the definition or unit of buffering strength. A fundamentally different issue not to be confused with these two is the question which is the pK_a that maximizes buffering strength when pH and total buffer concentration are fixed to particular values. Then, both β_{H^+} and the t,b,T,B system lead to the identical and correct conclusion. Namely, that weak acid is found to buffer most efficiently whose K_A is closest to the free H^+ ion concentration, i.e., for which $K_A=[H^+]_{free}$. There is no logical conflict with the previous conclusion that the point of maximum buffering is not at $[H^+]_{free}=K_A$.

Logarithmic transforms are readily dispensable for the quantitation of buffering action

Langmuir-like processes following the same mathematics as illustrated here for weak acids are encountered frequently in many disciplines outside acid-base chemistry (e.g. substrate binding in enzyme kinetics, or adsorption processes in membrane biochemistry). In these disciplines, it is common usage to employ linear units in order to measure and present the quantities involved, rather than using instruments and plots with logarithmical responsiveness or scales. Concepts analogous to "pH buffering" are not used in those disciplines, and peers would not easily accept propositions analogous to the ones that are notorious in acid-base chemistry. For instance, a chemist stating that "adsorption to a surface is most efficient if half of the binding sites are occupied" would be met with irritation, similar to an enzymologist stating that "changes of substrate concentration will affect an enzyme's activity most strongly around its K_M value". Only in the context of acid-base buffering, the perception of binding processes occurs almost

exclusively through –metaphorically speaking- logarithmical goggles. Just as one can learn to ride a motorbike wearing goggles with prisms that turn everything upside down, people in the acid-base field acquired the astounding ability to move along a hyperbola under their feet with a sigmoidal curve in their minds. The present article attempts, among other things, to cast the logarithmic goggles and move along a hyperbola a with a hyperbola in mind. Apparent difficulties upon switching from one view to the other are probably transient and certainly not a proof that the present logarithmic one should be more "natural".

References

1. DD Van Slyke: **On the measurement of buffer values and on the relationship of buffer value to the disociation constant of the buffer and the concentration of the buffer solution.** *J Biol Chem* 1922, **52:** 525-570.
2. E Neher: **The use of fura-2 for estimating Ca buffers and Ca fluxes.** *Neuropharmacology* 1995, **34:** 1423-1442.
