# Supplementary material for: The quantitation of buffering action II. Applications of the formal & general approach
Source: Theor Biol Med Model. 2005 Mar 16;2:9. doi: 10.1186/1742-4682-2-9 (PMC1079954; doi:10.1186/1742-4682-2-9)
Supplement: Additional File 4 — Other Non-Conservative Buffered Systems [file 1742-4682-2-9-S4.pdf]

Theoretical Biology and Medical Modelling

Research

The quantitation of buffering action. II. *Applications of the formal and general approach.*

Bernhard M. Schmitt

Supplement 4:

Other Non-Conservative Buffered Systems

Buffering of blood pressure variability

Variability of blood pressure and heart rate have recently raised great scientific interest. On the one hand, technical improvements facilitated and refined data acquisition and analysis. On the other hand, the various measures of variability obtained in these analyses were shown to be clinically useful indicators of cardiovascular risks, e.g. for hypertension or cardiac arrhythmia.

Analysis of blood pressure variability is instructive inasmuch it shows that a seemingly intuitive usage of the term "buffering" may in fact refer to phenomena that are actually not compatible with the semantic and quantitative core of the buffering concept.

Variability: a complex phenomenon that can be quantitated in multiple ways

In spite of the clinical usefulness of blood pressure variability measurements, the underlying mechanisms are not well understood. Little is known about the mechanisms that cause blood pressure variability, and little is also known about the mechanisms that tend to reduce, or "buffer", this variability. Variability buffering is a complicated phenomenon in several respects. A given mechanism may simultaneously increase variability in one frequency range, yet decrease it in another [1]. Moreover, a given mechanism, e.g. respiratory sinus arrhythmia, may buffer blood pressure variability under some conditions, but unexpectedly increase variability under other conditions [2]. The quantitation proper of blood pressure variability buffering, on the other hand, is complicated inasmuch there is no single universal measure of "variability", but more than a dozen indices that are used in parallel, including both "time domain" and "frequency domain" indices [3]. It is often difficult or impossible to compare separate studies because indices may have diverse intrinsic properties (e.g. the dependence on sampling time) and include particular weighting procedures (e.g. restriction to a particular frequency band).

Here, our focus is the question whether buffering terminology can be used at all in the context of variability. To deal with this question, we suspend

the problem of multiple competing measures of "variability" (or "noise" or "oscillations"), and simply carry out our argument under the assumption that there is at least one valid index of variability that can be expressed as a number on an interval scale.

Blood pressure variability resists formalization as "buffered system".

We then take variability as the output y of a transfer element, or as the dependent variable in a function. This raises the first problem, namely to identify the input or independent variable. For the buffering of free H^+ concentration, the independent variable was total H^+ concentration, and for the buffering of organ perfusion it was perfusion pressure. But against which potential disturbance is blood pressure variability buffered?

Experimentally, it is straightforward to change $[H^+]_{total}$ or ΔP in a controlled fashion in order to elicit and record the associated changes of $[H^+]_{free}$ or φ_I, respectively. Yet which graded stimulus might be used as a "titrant" to increase or decrease blood pressure variability in a given cardiovascular system? Such a titrant should probe the cardiovascular system without affecting buffering itself. Maneuvers such as baroreceptor denervation or pharmacological blockers, however, produce their effects on blood pressure variability predominantly by altering the level of buffering. In acid-base terms, this would be comparable to the removal or addition of buffer base – clearly not a straightforward approach to determine buffering strength of the original solution. In other words then, what is the source of blood pressure variability?

It is crucial to identify the independent variable because all available definitions of buffering strength, including our own one *(Buffering I)*, rest on the slope of a relation between two variables. Buffering strength cannot be quantitated solely from single point measurements of the output y, for instance, with and without baroreceptor deafferentiation, even though most studies of blood pressure variability follow such a design.

Quantitation of variability buffering presupposes knowledge about the system under conditions of zero buffering

Let us assume nonetheless that we had successfully identified such an independent variable, and subsequently established curves that describe blood pressure variability as a function of this variable with and without baroreceptor deafferentiation. We are then in a position to measure the two slopes $\tau'_1(x)$, $\tau'_2(x)$ and to tell whether nitric oxide blockade increases or decreases that slope, and by what factor. We are not able yet, however, to extract an absolute numerical value for buffering strength from this data.

The reason is that we do not know the slope of the corresponding curve in the completely unbuffered state, i.e., we do not know the derivative $\sigma'(x)$ of the sigma function $\sigma(x)$. Clearly, very different buffering strengths are compatible with a given slope $\tau'(x)$ when $\sigma'(x)$ is not constrained. In fact, without knowledge of the unbuffered response, there is no way to tell weak from strong or from even perfect buffering, or to distinguish inverting from non-inverting buffering, or "moderation" from "inverting amplification".

As directly apparent from the formal definition of the buffering parameters *(Buffering I)*, computing buffering strength requires knowledge of one buffered plus one unbuffered slope, or of one buffered slope plus the slope $\sigma'(x)$ of the sigma function. In contrast, the ratio of two different buffered slopes is not sufficient. The systems already mentioned in the previous examples may serve as a further illustration: there, it was impossible to determine buffering strength solely from several flow-flow relations (with different types or numbers of parallel "buffering tubes"; *see main text, Figure 2A-C*), or from several pressure-flow relations (with different types or numbers of serial "buffering tubes"; *see main text, Figure 2D*).

Taken together, current concepts of blood pressure variability buffering fail to specify two essentials, namely *1)* an independent variable and *2)* the relation between that independent variable and blood pressure variability under conditions of zero buffering. Without these, blood pressure variability

buffering strength cannot be quantitated in absolute terms, but only on a non-metric, ordinal scale at best.

"Variability buffering" – a dispensable term

The term "blood pressure variability buffering", in its currently usage, is fully replaceable by the synonymous term "blood pressure invariability", i.e., the opposite of blood pressure variability: The basic finding that "blood pressure variability has decreased" (as measured by one of the available indices of variability) may be rephrased into "blood pressure *invariability* has increased" or into "blood pressure variability buffering has increased", but the latter two statements do not produce any additional insight as compared to the original statement. In contrast, quantitative statements pertaining to "classical" buffering (e.g. H^+ buffering) are not simply rephrased statements about H^+ concentration: "H^+ buffering strength" and "H^+ concentration" are completely independent variables (analogous to the height vs. the steepness of a mountain), and buffering strength provides additional, independent information, namely about the slope of the relation between $[H^+]_{total}$ and $[H^+]_{free}$.

The confusion may stem from the fact that variability can be measured and expressed numerically and in various ways, whereas no numerical measure exists for "invariability". Analogously, there are numerical indices in mathematical statistics for "inaccuracy" (deviation from the true value) or "imprecision" (scatter around the empirical average), namely variance, standard deviation, or coefficient of variation, but there is no numerical index of "accuracy" or "precision". Therefore, inasmuch "blood pressure variability buffering" is exactly the same as "blood pressure invariability", there *can't* be a unit for it. This lack clearly shows that the meaning of the term "buffering" in the context of blood pressure variability does not overlap with the semantic core of the general, original buffering concept. We conclude that "blood pressure variability buffering" is a mainly metaphorical concept, and for fundamental reasons not compatible with a proper quantitative measure. The concept of blood pressure variability buffering is dispensable for the analysis of blood pressure variability, which itself is a very tangible concept. Abandoning the term "buffering" in the context of blood pressure variability could avoid confusion and make the original buffering concept clearer.

Electric amplifiers

The term "amplification" is most widely used in the context of electric current or voltage. Electric amplifiers can illustrate how the extent of "amplification" can be quantitated with the aid of our formal and general concept of buffering, and how amplification can turn seamlessly into moderation. Moreover, a certain type of amplifiers (voltage-to-current converters) are described by buffered systems that are similarly suited for the description of biomembranes with potentially non-linear current-voltage relationships. In this context, the notion of "rectification" can be endowed with a precise meaning and corresponding quantitative measures.

Voltage-to-voltage converters

Consider the static properties of electronic amplifiers with linear amplification characterstics. For instance, most feed-back amplifiers used in electrophysiology transform an input voltage V_{in} into an output voltage V_{out} that is simply the product of the variable input and a constant gain A:

$$V_{out} = V_{in} \times A.$$

We take input voltage V_{in} as the independent variable x, and output voltage V_{out} as the transfer function $\tau(x)$. As „unbuffered system response", we posit that condition under which input voltage equals output voltage; this situation is obtained with the gain set to $A=1$. Assuming the absence of any „offsets", the corresponding buffered system is given as

$$\begin{pmatrix} x \\ \tau(x) \\ \beta(x) \end{pmatrix} \leftrightarrow \begin{pmatrix} V_{in} \\ V_{out} \\ V_{in} - V_{out} \end{pmatrix}.$$

This buffered system is dimensionally homogeneous (independent and dependent variables are voltages) and conservative inasmuch as $\tau'(x)+\beta'(x)=1$. By

setting gain A to various values, this system can replicate all possible types of linear buffering behavior, including „negative buffering“: A gain $A>0$ produces non-inverting buffering, a gain $A<0$ inverting buffering. A gain with $|A|<1$ produces moderation, and a gain with $|A|>1$ amplification („negative buffering“). These results agree with intuition: when the output is greater than the input, it appears reasonable to say that the input was „amplified“, or, when it is smaller, that it was moderated.

Voltage-to-current converters

Other types of amplifiers convert the input voltage into a proportional *current* output I_{out} (e.g., certain feed-back amplifiers for two-electrode voltage clamp by NPI electronic, Tamm, Germany). The relation between input voltage and output current can be visualized using an I-V plot. The proportionality factor in this case is not any more a dimensionless gain that, when set to a value of $A=1$ (i.e., input=output), can define a corresponding „canonical“ buffered system. Rather, the output is related to the input via a conversion factor K that has the dimensions of a conductance ($[G°]=[\text{current}]/[\text{voltage}]$). As such, it cannot assume the „canonical“ dimensionless value of 1, and there is also no such thing as a „unit conductance“ – every attempt of standardization by adopting a specific unit must remain arbitrary. Accordingly, one cannot distinguish in a natural way between moderation and amplification solely from a given combination of input voltage and output current. If the conductance G^0 is known, however, it can be posited as the system response in the unbuffered state, and thus define a formally correct buffered system (assuming again zero offsets at $x=0$):

$$\begin{pmatrix} x \\ \tau(x) \\ \beta(x) \end{pmatrix} \leftrightarrow \begin{pmatrix} V_{in} \\ I_{out} \\ G^0 \times V_{in} - I_{out} \end{pmatrix}$$

The parameters t, b, T, and B computed for such a system are all dimensionless numbers.

Biological membranes as voltage-to-current converters

The conversion of an input voltage into a linearly related output current can be represented by a generic transfer element inside a „black box“. However, the same behavior of the transfer element that is produced by the electronic circuit of a voltage-to-current converter can be produced by a fixed conductance $g°_m$, e.g. by a simple ion channel with a perfectly linear ohmic conductance and an open probability of unity in a biomembrane. It is reasonable to define as „zero buffering“ that condition under which membrane slope conductance $g°_m$ equals the number N of channels times the unitary channel conductance g:

$$g°_m = N \times g \, .$$

This is sufficient to formulate this bioelectrical system as a buffered system:

$$\begin{pmatrix} x \\ \tau(x) \\ \beta(x) \end{pmatrix} \leftrightarrow \begin{pmatrix} V_m \\ I_m \\ g°_m \times V_m - I_m \end{pmatrix}$$

Here, the conversion factor K has the form of a “standard” conductance $g°_m$, analogous to the conversion factor of the voltage-to-current converting amplifier (ignoring the fact that asymmetrical ionic conditions may shift the I-V curve to more negative or positive voltages).

A quantitative measure of rectification

Most biomembranes, however, do not behave in a simple and linear way as electronic amplifiers or perfectly ohmic ion channels. Rather, they display pronounced non-linear behavior. Individual ion channels, for instance, may exhibit conductances that are ohmic only within a certain voltage range, and decrease progressively towards zero beyond a certain voltage. This behavior, known as “rectification”, has important physiological implications. Such a biomembrane cannot be characterized by a single fixed conductance $g°_m$, but only by a variable conductance $g(V_m)$ that is a function of membrane potential.

We can interpret, however, the membrane current I_m from the range where g_m is constant and

independent of V_m as the "normal", unbuffered response of the biomembrane to the membrane potential V_m, and regard, according to the usual definition, its deflection from a straight line as "rectification" (or "buffering"). The conversion factor K of the corresponding buffered system is provided by membrane conductance within its linear range, symbolized by g^*:

$$\begin{pmatrix} x \\ \tau(x) \\ \beta(x) \end{pmatrix} \leftrightarrow \begin{pmatrix} V_m \\ I_m \\ g^*_m \times V_m - I_m \end{pmatrix}$$

Thus, the relationship between V_m and the current I_m together with a constant "virtual" slope conductance g^*_m considered to represent a situation with zero rectification allow to calculate the four buffering parameters t, b, T, and B. The buffering coefficient b and the buffering ratio B in this case constitute quantitative measures of rectification at a given membrane potential V_m. The degree of rectification varies widely between ion channels, and scientists verbally distinguish "strong" and "weak" rectification. So far, however, there has been no quantitative measure of the degree of rectification.

Properties that contribute to threshold behavior and oscillations can be identified by the buffering parameters t, b, T, or B

The I-V relationships of membranes that contain rectifying conductances often contain sections where the current is actually *decreasing* with increasing voltage, i.e., sections with negative slope conductances g_m. Therefore, the entire I-V curve may intersect the voltage axis (i.e., $I_m = 0$) more than once, either falling or rising ($g_m<0$ or $g_m>0$, respectively), and the intersections form unstable and stable equilibrium points. Under certain conditions, such systems may either display threshold behavior or behave as oscillators [4], two remarkable properties that are of great physiological importance, e.g. for cardiac pacemaker cells, pulsatile hormone release from endocrine cells, sleep-wake cycle, and various neuronal networks.

The conditions for unstable and stable equilibrium points of passive conductors are: ($I_m=0$ and $g_m<0$) and ($I_m=0$ and $g_m>0$), respectively. We can make this concept independent from the context of electrical circuits and express it in a general form by replacing the specific quantities I_m and g_m by the general variables and parameters of non-conservative, dimensionally heterogeneous buffered systems. Then, we obtain the sufficient conditions for an unstable equilibrium point as:

unstable equilibrium $\Leftrightarrow$ $\tau(x)=0 \wedge t<0$

and the condition for a stable equilibrium point:

stable equilibrium $\Leftrightarrow$ $\tau(x)=0 \wedge t>0$.

The presence of stable or unstable equilibrium points is necessary, but not sufficient to give rise to threshold or oscillating behavior, however. The buffered system needs to be combined into a network with the respective equivalents of an electrical energy source and energy storage device. Provided these criteria are fulfilled, even simple physico-chemical buffered systems may display threshold or oscillating behavior. The requirements presented above provide universal criteria for unstable and stable equilibrium points in very diverse biological or technical systems. This generalization is made possible by the formal and general nature of our approach, analogous to our generalization of the "isohydric principle" *(Buffering I)*.

References

1. A Just, U Wittmann, B Nafz, CD Wagner, H Ehmke, HR Kirchheim, PB Persson: **The blood pressure buffering capacity of nitric oxide by comparison to the baroreceptor reflex.** *Am J Physiol* 1994, **267:** H521-H527.
2. JA Taylor, DL Eckberg: **Fundamental relations between short-term RR interval and arterial pressure oscillations in humans.** *Circulation* 1996, **93:** 1527-1532.
3. Task force of The European Society of Cardiology: **Heart rate variability: standards of measurement, physiological interpretation, and clinical use.** *Europ Heart J* 1996, **17:** 354-381.
4. TF Fischer Weiss: *Cellular Biophysics*. Cambridge, MA, USA: MIT Press; 1997.
